# A Unique Collection of Palaeolithic Painted Portable Art: Characterization of Red and Yellow Pigments from the Parpalló Cave (Spain)

**DOI:** 10.1371/journal.pone.0163565

**Published:** 2016-10-12

**Authors:** Clodoaldo Roldán García, Valentín Villaverde Bonilla, Isabel Ródenas Marín, Sonia Murcia Mascarós

**Affiliations:** 1 Institute of Material Science (ICMUV), University of Valencia, Paterna, Valencia, Spain; 2 Department of Prehistory and Archaeology, University of Valencia, Valencia, Spain; Universidade do Algarve, PORTUGAL

## Abstract

In this work we analyze the pigments used in the decoration of red and yellow motifs present in the portable art of the Parpalló Cave (Gandía, Spain), one of the most important Palaeolithic sites in the Spanish Mediterranean region. Energy dispersive X-ray fluorescence spectrometry (EDXRF) and spectrophotometry in the visible region (CIEL*a*b*color coordinates and spectral reflectance curves) were used to perform *in situ* fast analyses of the red and yellow motifs with portable equipment and to characterize their elemental composition and their colorimetric perception, respectively. According to the elemental composition, the intensity of the fluorescence iron signals in red and yellow motifs are higher than average values in the rock substrates. As expected, red motifs possess high values of the chromatic coordinate a* and yellow motifs possess high values of b*. This characterization was complemented with FT-IR analyses of microsamples detached from the red and yellow colored zones of a small set of plaquettes. Our results show that the artists used red and yellow pigments in the decoration likely derived from natural iron oxides as hematite and goethite.

## Introduction

The portable art of the Parpalló Cave (Gandía, Valencia, Spain) is one of the reference collections for the study of the Palaeolithic Art in Europe [1]. The site, located in the central Mediterranean region of the Iberian Peninsula, has provided a large collection of decorated plaquettes that is unique and exceptional for various reasons, such as the fact that the collection covers the entire archaeological sequence from the Gravettian to the Magdalenian periods (26000–11000 years BP) and that it enables the identification of a wide variety of engraved and painted motifs (zoomorphs and non-figurative motifs or signs) decorated with black, red, and yellow pigments [1–3]. The painted plaquettes of the Parpalló collection include 81 zoomorphic motifs, 194 non-figurative motifs or sings (diverse geometric forms, spots and bands), and about two thousand plaquettes with undefined non-figurative motifs which, in some cases could be the result of pigment processing or accidental pigmentation by contact with colorants presents in the same archaeological and stratigraphic level.

The identification and characterization of the prehistoric pigments are usually made from small samples which have been analyzed by means of a variety of analytic techniques: scanning electron microscopy with microanalysis (SEM-EDS) [4–6], X-ray diffraction (XRD) [7, 8], Raman spectroscopy [8, 9], particle induced X-ray emission (PIXE) [10], inductively coupled plasma mass spectrometry (ICP-MS) [7, 11]. However, due to the remarkable archaeological, historical and cultural value of the plaquettes from the Parapalló collection, it is mandatory that a minimally invasive analytical protocol preserves the integrity of the pigments, avoiding their movement outside the museum environment and using portable and non-aggressive analytical techniques in order to perform in situ and non-destructive measurements.

Recently, much effort has been made to improve the instrumentation for in situ and non-destructive analyses of the archaeological patrimony. In this sense, the energy dispersive X-ray fluorescence spectroscopy (EDXRF) is a valuable technique extensively and successfully applied to the study and characterization of the prehistoric rock art [12–18]. On the other hand, colorimetry and spectrophotometry provide a powerful tool for a better understanding of a wide range of color related problems, such as the identification and characterization of pigments and the evolution of the color proprieties that can occur with the age [19, 20].

This contribution complements a previous scientific study made by the authors and centered on the characterization of black pigments used in the decoration of the Parpalló plaquettes [16]. With this new study we extend the characterization of pigments to the entire color range identified in the collection, incorporating the analyses of red and yellow pigments present in the painted motifs of this exceptional Palaeolithic collection of portable art.

## Materials and Methods

### The portable art of the Parpalló Cave with red and yellow pigments

The portable art collection of Parpalló consists in 5612 calcareous plaquettes with 6245 engraved and/or painted surfaces (some pieces present decoration on the both faces) which span the entire Palaeolithic sequence from the Gravettian to the Upper Magdalenian. In Table 1 we show the number of plaquettes of the Parpalló collection divided into the different archaeological periods classified by painted motifs. The number of plaquettes and the diverse decoration techniques show significant variations along the different levels of the stratigraphic sequence. On the other hand, the distribution of the plaquettes in spatial terms is not clearly defined [1].

**Table 1:** Chronological and thematic distribution of the plaquettes from the Parpalló collection.

	G	LS	AMS	RMS	US	SGI	SGII	SGIII	AMA	AMB	UM
**Number of Plaquettes**	7	154	326	529	558	344	218	353	323	671	440
**Number of Surfaces**	13	193	402	655	696	419	245	415	416	883	557
**Number of Zoomorphs**	7	63	104	50	49	55	35	30	46	85	70
**Number of Painted Zoomorphs**	2	14	17	10	6	5	3	7	0	1	2
**Number of Signs**	11	155	240	465	444	208	112	146	293	773	466
**Number of Painted Signs**	3	21	22	46	36	18	7	9	6	9	7
**Number of Painted Surfaces**	3	31	101	219	252	163	98	229	113	152	112
**% Painted Signs**	27.3	13.5	9.2	9.9	8.1	8.6	6.2	6.2	2.1	1.2	1.5
**% Painted Zoomorphs**	28.6	22.2	16.3	20.0	12.2	9.1	8.6	23.3	0.0	1.2	2.8

G: Gravettian (28000–21000 BP). LS: Lower Solutrean (21000–20500 BP). AMS and SMS: Ancient and Recent Middle Solutrean (20500–20000 BP). US: Upper Solutrean (2000–19500 BP). SG-I, SGII and SGIII: Solutreo-Gravettian I, II and III (19500–17000 BP). AM: Ancient Magdalenian A and B (17000–145000 BP). UM: Middle and Upper Magdalenian (14.500–12.000). Chronologies are only indicatives.

Actually, the collection of the plaquettes from the Parpalló Cave is stored in the bonded warehouse of the Museo de Prehistoria de Valencia (C/ Corona, 36. ZIP Code 46003. Valencia, Spain) and a small selection of them are on the exhibition rooms of this museum. From the archaeological point of view, the artistic sequence of the Parpalló plaquettes provides useful information to characterize the evolution of the Palaeolithic art of the Iberian Mediterranean region from a thematic, stylistic and technical perspective. Given the scarcity of Solutrean portable art in Cantabrian, Aquitania and Pyrenean regions, the Parpalló collection is especially important to evaluate the artistic characteristics of this period. In this sense, it is remarkable the high number of good quality depictions in form of engraved and painted zoomorphs and sings that are found in the Solutrean levels. A stylistic comparison between these motifs and the parietal art motifs open the possibility to establish a chronological reference to intervene at the ordination of the Palaeolithic parietal art of the Iberian Peninsula.

The pictorial motifs of the Parpalló collection were the result of a combination of different techniques and chromatic variations: linear motifs (fine and broad-lined) and extended paintings (painted surfaces), simultaneous presence of engraved and painted designs, monochrome and, in some cases, bichrome representations. In a significant number of plaquettes the pigment covers the whole surface (sometimes only on one side and others on both sides). In these plaquettes we cannot dismiss the hypothesis of their use for processing the raw pigment and therefore they should not be considered as expressions of portable art. For these reasons we have focused our attention on the figurative themes and well-defined signs, although we have also included analysis of plaquettes with indeterminate signs, stains or marks to compare the analytical results of different pictorial motifs.

**Fig 1:**
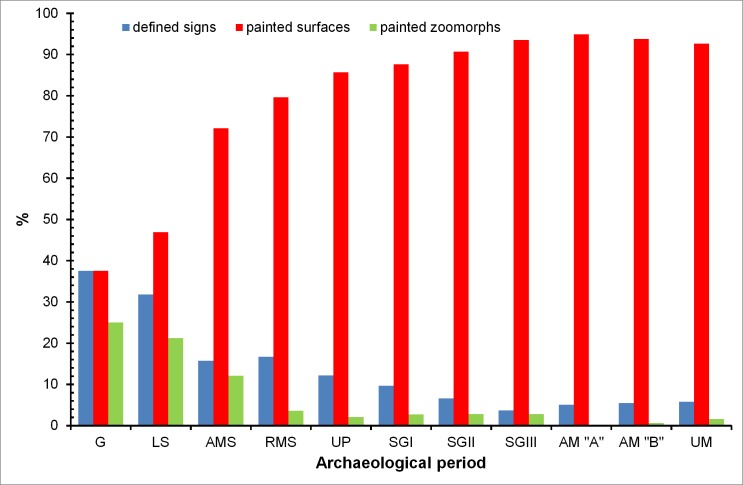
Percentage of painted signs, painted surfaces and painted zoomorphs present in the Parpalló collection. Percentage values have been calculated with respect to the total painted plaquettes from each period.

The percentage of the Parpalló plaquettes decorated with zoomorphic motifs and well-defined signs (calculated with respect to the total number of painted plaquettes in each period) present a decreasing trend from the Solutrean to the Magdalenian period (Fig 1). However, the greatest number of these motifs appears concentrated in the Solutrean levels (Table 1). Moreover, the percentage of plaquettes with painted surfaces without defined pictorial motifs present an increasing trend from the Gravettian to the Magdalenian (Fig 1) suggesting that these plaquettes could be related to the pigment processing. Therefore, precautions should be taken in any artistic or stylistic evaluation of this kind of plaquettes.

**Fig 2:**
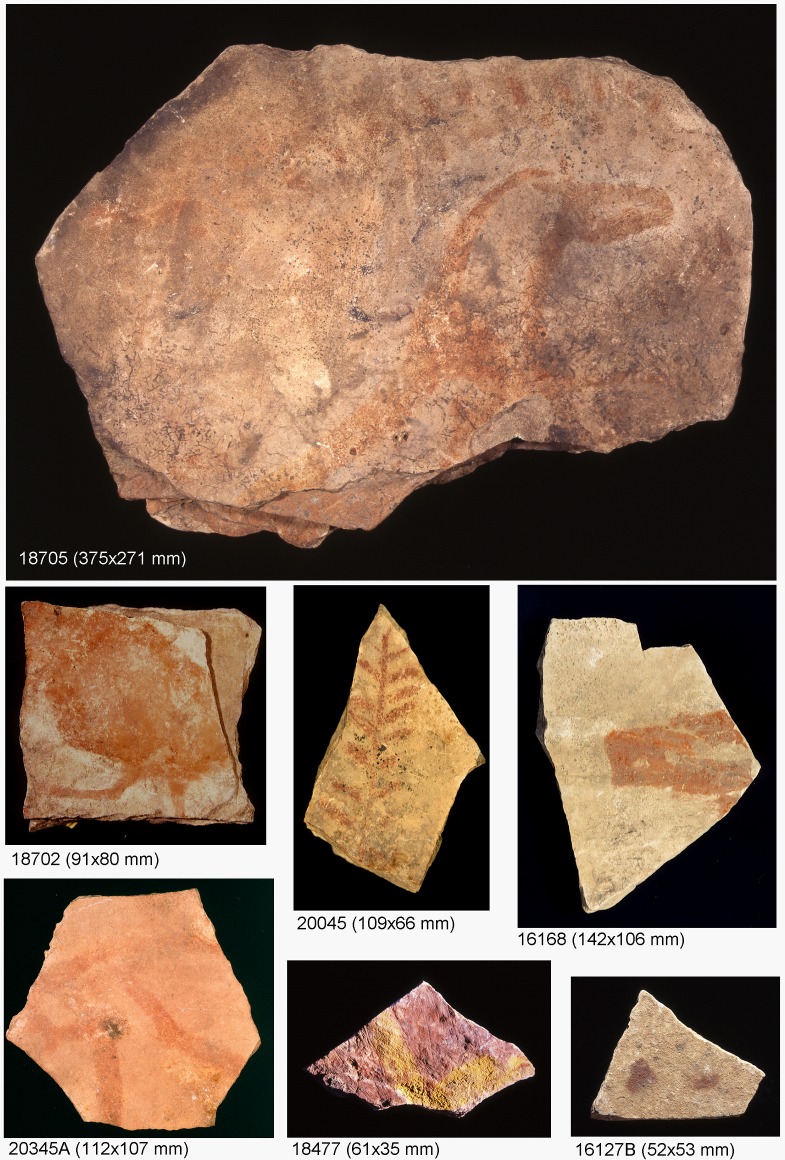
Selection of analyzed plaquettes from the Parpalló collection showing red and yellow motifs. The number under the picture corresponds to the identification code.

The analyzed plaquettes presented in this work cover a wide chronological interval from the Lower Solutrean (LS: 21500–20500 BP) to the Upper Magdalenian (UM: 13500–12000 BP) and present a great variety of decorative motifs: zoomorphs (with identified species as aurochs, cervids, horses, ibex and legs and snouts from non-identified and fragmentary animals), and signs as penniforms or ramiforms, pectiforms, extended paints, lines and dots. Also, nonfigurative motifs are present in the surface of the plaquettes in colored areas with well defined contours (delimited painted surfaces) or in form of colored areas with diffuse contours (painted surfaces) that sometimes cover the whole of the plaquette. There are no anthropomorphic motifs in the analyzed plaquettes. Different paint techniques are present in the surface of the plaquettes: monochrome uniform paints, monochrome uniform paints combined with outline engraving, linear paint stroke, and linear paint stroke combined with outline engraving. The characteristics of the analyzed plaquettes and reference codes for their identification are included in S1 and S2 Tables and images of a selected set of them are shown in Fig 2. These reference codes are the same reference codes used in the internal identification of the Museo de Prehistoria de Valencia. All necessary permits were obtained for the described study, which complied with all relevant regulations. Analyses included in this paper have been made under permission of the Dr. Helena Bonet Rosado, Director of the Museo de Prehistoria de Valencia (Eixida Num. 164/16).

A set of 73 sides with red motifs from 67 plaquettes and 15 sides with yellow motifs from 14 plaquettes have been analyzed by EDXRF spectrometry and spectrophotometry (colorimetry) at the Museo de Prehistoria de Valencia by means of portable instrumentation according to a non-invasive analytical protocol. Both non-invasive EDXRF and colorimetric analyses were performed on the surface of the plaquettes with no preparation or sampling. These analyses were carried out on flatter areas of the pigmented and non-pigmented zones of each plaquette in order to compare the analytical results from the pigments and the unpainted rock supports. This comparison can help us to identify deliberately applied pigments or surface colorations related with geogenic, biogenic or post-depositional processes. Additionally, detached micro-samples from a selected number of plaquettes with red and yellow pigments were taken to perform Fourier transform infrared reflectance (FT-IR) analyses.

### Color analysis

Colorimetry and visible spectrophotometry are crucial techniques in the documentation of cultural heritage objects. Colorimetry includes the wavelength intensities of standard illuminants selected by the *Commission Internationale de l’Eclairage* (CIE) [21] and provides a standard procedure to describe a color stimulus in terms of defined illuminants and a defined standard observer. Visible spectrophotometry is a non-invasive technique which measures the amount of light reflected or transmitted by a material at individual wavelengths of the spectrum. The relative percent of electromagnetic power reflected or transmitted at each wavelength is called spectral curve and is unique for various pigments and thus can be helpful to identify iron oxides [22–24].

Spectrophotometry measurements have been recorded as diffuse reflectance spectral curves in the visible wavelength range (360–740 nm) with a pitch of 10 nm using a MINOLTA CM2600D equipment. Trichromatic color coordinates were measured within the CIE L*a*b* space adjusted to the experimental conditions. Reflectance spectral curves and color measurements were taken on points of the red and yellow painted areas and on unpainted areas of the surface of the plaquettes with a 3 mm diaphragm, specular component excluded, standard lighting D65, 10° observer angle, 3-flash mode auto-averaging and lit with no UV components pulse xenon flash light source. Color differences between pigments and unpainted rock surfaces were evaluated computing color differences from the CIE L*a*b* coordinates with the Euclidean distance CIE76 formula ΔE^*^_ab_ [21].

### EDXRF spectrometry

In order to estimate the elemental composition of the red and yellow pigments, a home-made and tailored to user-specific needs portable energy dispersive X-ray fluorescence spectrometer was used (Roldán et al., 2013). The spectrometer is equipped with a Peltier cooled semiconductor Si-PIN detector (AMPTEK XR-100CR, FWHM = 175 eV @5.9 keV) and a low power X-ray transmission tube (Eclipse-II, silver anode) operating with an excitation potential of 30 kV and an intensity of 0.004 mA. An aluminium pinhole collimates the X-ray beam on a sample surface of about 70 mm^2^. Data acquisition was controlled by a “pocket” multichannel buffer board. X-ray tube and detector were fitted on a mechanical device with an excitation-detection geometry of 45° and about 2 cm sample-detector distance. EDXRF measurements were performed with acceptable statistic for 180 s and processed with the PyMCA code [25]. Light elements with Z<14 were not detected.

Net areas of elements present in the fluorescence spectra were normalized to the total counts of the spectra to minimize the X-ray tube intensity fluctuations and geometrical effects derived from irregularities from the surface of the samples. The variability of the paint layers (color gradients, thickness, surface roughness, etc.) is incompatible with a quantitative analysis derived from the EDXRF data and, consequently, we performed a qualitative analysis based on the comparison of the normalized net areas of the fluorescence peaks.

### FT-IR spectroscopy

Detached microsamples with red and yellow colored motifs (see the FT-IR Analysis section) and the underlying rocks were analyzed by means of a Bomen ABB FTLA 2000 Fourier Transform Infrared Spectrometer equipped with a Spectra-Tech Analytical Plan Microscope with the diamond cell as sample holder. FTIR spectra were collected in the 4000–350 cm^-1^ and 4000–720 cm^-1^ wavelength range with a resolution of 4 cm^-1^.

## Results and Discussion

### Colorimetry

**Fig 3:**
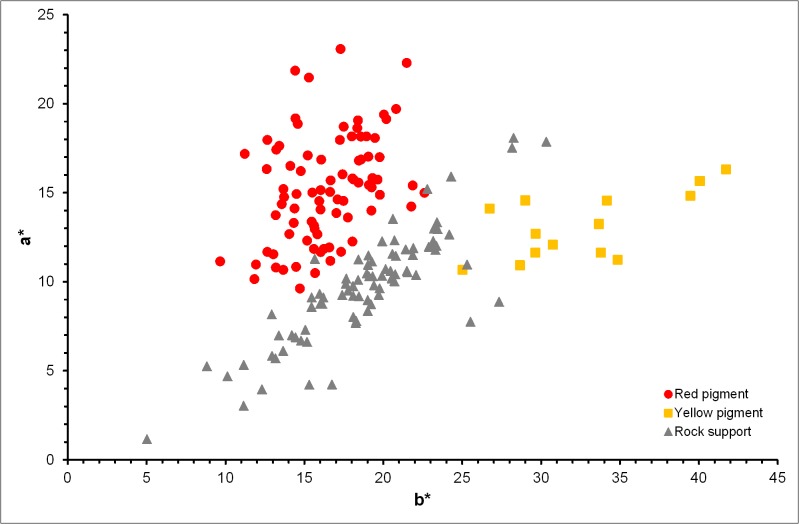
CIEL*a*b* chroma diagram for red and yellow pigments and rock supports.

After the calibration performed in the spectrophotometer, three readings were taken on each analyzed point of the red and yellow motifs and on the unpainted roc surface of the plaquettes. From the average values of these readings, the CIEL*a*b* coordinates were calculated and the spectral curves were registered. Tables 2 and 3 give the colorimetric L*a*b* coordinates and the dominant wavelength for 191 measurement points (82 from red motifs, 14 from yellow motifs and 95 from the rock supports). Red and yellow pigments from the Parpalló plaquettes had positive values of the colorimetric coordinates with L* ranging from 24.81 to 50.32 for red and from 50.18 to 60.08 for yellow; a* ranging from 9.62 to 23.08 for red and from 10.67 to 16.31 for yellow; b* ranging from 9.66 to 22.61 for red and from 25.03 to 41.72 for yellow. The CIEL*a*b* coordinates of the rock supports present a noticeable differentiation with respect to the pigments with L* ranging from 35.26 to 64.80, a* ranging from 1.16 to 18.08 and b* ranging from 5.03 to 30.33. Fig 3 shows that red and yellow pigments can be differentiated in the a*-b* color space. Yellow pigments are separated from red pigments by their significantly higher yellowness (b*) and lower redness (a*). For pure hematite the a* and b* values ranging from 15 to 30 and from 2 to 30, respectively, whereas for pure goethite the a* and b* values ranging from 5 to 15 and from 22 to 48, respectively [22].

**Table 2:** L*a*b* colorimetric coordinates for red motifs from the plaquettes of the Parpalló Cave, dominant wavelength (λ_D_) and color differences ΔE*ab between underlying rock supports and red motifs.

Plaquette	Face	L*	a*	b*	λ_D_ (nm)	ΔE
**16007**		**40.40**	**16.33**	**12.61**	**594.08**	**21.57**
**16024**		**37.19**	**15.31**	**19.26**	**586.84**	**22.14**
**16112**		**47.25**	**14.89**	**19.78**	**585.42**	**4.36**
**16112**		**38.79**	**10.67**	**13.66**	**585.25**	**9.28**
**16119**	**B**	**43.91**	**16.51**	**14.11**	**592.09**	**14.32**
**16120**		**42.73**	**12.26**	**18.04**	**584.03**	**11.47**
**16126**		**44.16**	**15.57**	**18.42**	**587.15**	**13.00**
**16127**	**A**	**43.26**	**18.17**	**18.93**	**589.41**	**17.14**
**16127**	**B**	**38.86**	**11.85**	**15.60**	**585.23**	**12.98**
**16128**		**39.93**	**11.93**	**16.58**	**584.63**	**10.77**
**16157**		**40.63**	**10.49**	**15.67**	**583.59**	**11.13**
**16167**		**44.53**	**13.38**	**15.46**	**586.91**	**12.89**
**16168**		**40.75**	**19.71**	**20.81**	**589.80**	**18.47**
**16169**		**41.24**	**15.15**	**16.03**	**588.64**	**6.60**
**16170**		**45.48**	**16.81**	**18.45**	**588.29**	**6.74**
**16171**		**42.54**	**14.55**	**17.47**	**586.83**	**18.04**
**16245**		**41.02**	**13.86**	**17.02**	**586.46**	**17.31**
**16246**		**45.33**	**17.03**	**19.05**	**588.11**	**15.14**
**16246**		**29.79**	**12.68**	**14.04**	**588.22**	**30.81**
**16322**		**46.02**	**14.63**	**17.10**	**586.97**	**8.01**
**16329**		**49.42**	**17.00**	**19.77**	**587.38**	**7.97**
**16329**		**43.60**	**12.67**	**15.82**	**585.82**	**9.64**
**16406**	**A**	**45.95**	**10.97**	**11.93**	**587.10**	**9.13**
**16406**	**B**	**48.74**	**15.74**	**19.63**	**586.28**	**14.20**
**16607**		**34.26**	**16.86**	**16.06**	**591.19**	**21.92**
**16735**		**38.30**	**14.93**	**14.50**	**589.90**	**17.18**
**16753**		**44.26**	**15.83**	**19.31**	**586.83**	**8.33**
**17111**		**45.88**	**19.14**	**20.19**	**589.28**	**14.08**
**17251**		**50.32**	**15.44**	**19.09**	**586.24**	**17.59**
**17279**		**43.16**	**10.84**	**14.47**	**584.65**	**10.52**
**17316**		**38.20**	**18.87**	**14.58**	**594.91**	**22.07**
**17318**		**38.05**	**18.08**	**19.47**	**589.39**	**12.09**
**17375**	**A**	**44.97**	**18.64**	**18.35**	**590.16**	**10.05**
**17375**	**B**	**38.12**	**11.15**	**9.66**	**590.76**	**10.29**
**17375**	**B**	**42.98**	**17.97**	**17.25**	**590.56**	**9.64**
**17416**		**41.59**	**13.31**	**14.32**	**587.96**	**8.65**
**17419**		**41.46**	**15.76**	**18.10**	**587.74**	**10.36**
**17420**		**42.82**	**15.70**	**16.66**	**588.63**	**9.73**
**17617**	**B**	**45.45**	**15.81**	**18.04**	**587.57**	**16.32**
**17740**		**39.33**	**17.64**	**13.40**	**594.79**	**19.71**
**17787**		**24.81**	**18.17**	**18.00**	**592.43**	**28.22**
**17828**		**48.51**	**12.31**	**15.17**	**585.67**	**17.13**
**17847**		**36.88**	**23.08**	**17.29**	**597.15**	**34.41**
**17956**	**B**	**39.48**	**11.85**	**16.23**	**584.77**	**13.92**
**17960**		**43.82**	**11.69**	**17.32**	**583.8**	**10.25**
**18007**		**36.80**	**17.97**	**12.65**	**596.79**	**26.00**
**18009**	**A**	**35.74**	**21.47**	**15.28**	**597.85**	**22.00**
**18127**		**45.44**	**14.06**	**16.02**	**587.19**	**15.49**
**18227**		**37.33**	**21.86**	**14.41**	**599.40**	**21.56**
**18465**		**38.59**	**12.97**	**15.65**	**586.60**	**26.84**
**18477**		**41.47**	**13.16**	**15.61**	**586.68**	**4.74**
**18700**		**33.78**	**19.39**	**20.04**	**590.83**	**15.45**
**18700**		**34.81**	**14.53**	**15.94**	**588.48**	**10.81**
**18702**		**30.79**	**18.16**	**18.57**	**590.97**	**22.39**
**18704**	**A**	**45.64**	**14.23**	**21.77**	**583.97**	**6.07**
**18704**	**A**	**37.47**	**11.18**	**16.64**	**583.93**	**13.76**
**18705**		**39.89**	**14.00**	**19.24**	**585.25**	**15.33**
**18705**		**39.49**	**9.62**	**14.71**	**583.23**	**16.29**
**18705**		**42.16**	**13.61**	**17.75**	**585.61**	**13.28**
**18716**		**40.76**	**16.22**	**14.77**	**591.14**	**10.32**
**18728**	**A**	**47.15**	**15.00**	**22.61**	**585.57**	**15.14**
**18728**	**A**	**45.31**	**10.80**	**13.19**	**584.19**	**4.41**
**18788**	**A**	**38.15**	**13.74**	**13.18**	**589.79**	**21.34**
**18788**	**A**	**41.18**	**10.15**	**11.83**	**586.20**	**19.02**
**18788**	**B**	**42.03**	**11.55**	**13.03**	**586.91**	**7.20**
**18879**		**39.82**	**19.18**	**14.43**	**595.39**	**18.01**
**18880**		**41.67**	**15.05**	**16.63**	**588.04**	**10.94**
**18885**	**B**	**44.65**	**11.68**	**12.65**	**587.35**	**7.56**
**18935**		**41.48**	**17.19**	**11.22**	**597.84**	**19.77**
**18938**	**B**	**37.34**	**22.30**	**21.48**	**592.39**	**17.82**
**19336**		**39.38**	**15.01**	**15.50**	**589.04**	**10.30**
**19433**		**43.00**	**19.07**	**18.39**	**590.78**	**21.08**
**19650**	**B**	**43.73**	**16.04**	**17.41**	**588.36**	**5.13**
**19679**	**A**	**40.00**	**15.21**	**13.67**	**591.14**	**24.01**
**19683**	**B**	**43.83**	**18.71**	**17.49**	**591.13**	**12.20**
**19857**	**B**	**40.21**	**14.12**	**14.37**	**588.97**	**14.87**
**19864**		**43.42**	**16.87**	**18.59**	**588.38**	**28.01**
**20003**		**35.48**	**14.36**	**13.56**	**590.41**	**12.55**
**20004**	**A**	**37.51**	**17.10**	**15.21**	**592.08**	**12.22**
**20004**	**B**	**35.20**	**17.42**	**13.21**	**595.15**	**14.65**
**20045**		**40.57**	**14.76**	**13.71**	**590.40**	**15.09**
**20345**	**A**	**45.45**	**11.67**	**16.03**	**584.45**	**11.43**
**20345**	**B**	**50.27**	**15.41**	**21.86**	**584.69**	**10.51**

**Table 3:** L*a*b* colorimetric coordinates for yellow motifs from the plaquettes of the Parpalló Cave, dominant wavelength (λ_D_) and color differences ΔE*ab between underlying rock supports and yellow motifs.

Plaquette	Face	L*	a*	b*	λ_D_ (nm)	ΔE*ab
**16406**	**A**	**60.08**	**10.93**	**28.66**	**578.85**	**19.61**
**16607**	**A**	**54.41**	**14.83**	**39.47**	**579.70**	**21.76**
**17375**	**A**	**50.18**	**14.11**	**26.74**	**581.95**	**12.91**
**17617**		**53.35**	**14.57**	**29.00**	**581.54**	**11.74**
**17742**	**A**	**56.25**	**11.64**	**29.64**	**579.24**	**13.03**
**17956**	**A**	**56.78**	**14.56**	**34.17**	**580.25**	**21.39**
**18005**		**54.29**	**12.08**	**30.75**	**579.38**	**17.39**
**18009**		**53.39**	**11.23**	**34.85**	**578.27**	**20.90**
**18037**		**51.94**	**16.31**	**41.72**	**580.38**	**21.98**
**18206**		**53.50**	**11.63**	**33.79**	**578.66**	**13.55**
**18465**		**51.67**	**12.70**	**29.65**	**580.09**	**18.05**
**18477**		**50.59**	**13.24**	**33.66**	**579.76**	**18.72**
**18885**	**A**	**55.07**	**10.67**	**25.03**	**579.56**	**9.93**
**18929**		**54.77**	**15.66**	**40.06**	**580.06**	**17.41**

According to our data, the color differences ΔE*ab between the painted motifs and the underlying rock supports was significantly for all of the plaquettes (above the 4 ΔE units). Appreciable variability were found in ΔE*ab values from the red motifs (Table 2), the most distinct color differences correspond to the painted surface of the plaquette 16246 (ΔE: 30.81) and the least to the zoomorphic motifs of the plaquettes 16112 and 18728 (face A) (ΔE: 4.6 and 4.41, respectively). Minor variability in color difference was found in yellow motifs (Table 3): values ranging between 9.93 (delimitated painted surface of the plaquette 18885 (face B)) and 21.98 (painted surface of the plaquette 18037 (face B)).

**Fig 4:**
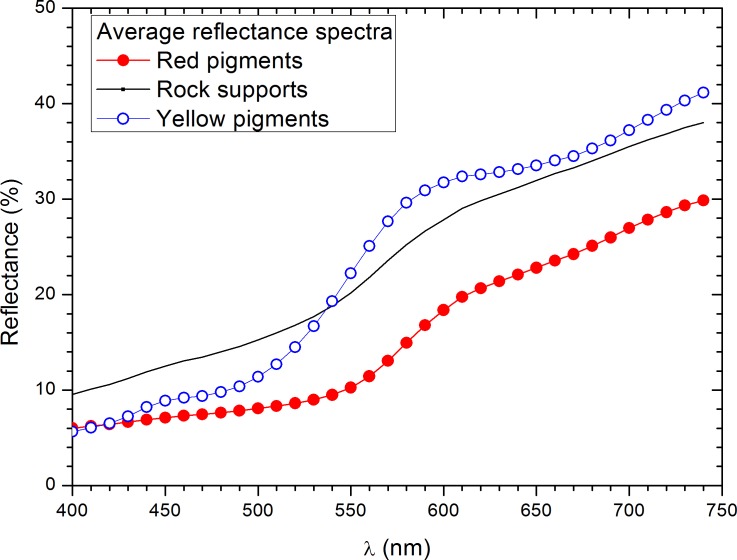
Average reflectance curves of the red and yellow pigments and rock supports.

The colors of the iron oxides result from several ligand-field transitions within the visible wavelength range. Iron oxides weakly reflecting in the ultraviolet region and are strongly reflecting in the visible region. Average reflectance spectra from red and yellow motifs and rock supports are shown in Fig 4. Yellow motifs show weak absorption bands that correspond to the characteristics bands of the goethite (649, 480 and 434 nm) within the visible wavelength range [22, 26]. Absorption bands of the hematite (649, 529, 444, 404 and 380 nm) or other red iron oxides are unappreciated in the reflectance spectra of the red motifs, probably due to the proportion and nature of other red iron oxides or minerals associated with the used red raw materials [26]. Red and yellow motifs could be differentiated from the rock supports in function of their respective reflectance spectra in the visible region. Red motifs show lower reflectance values than the yellow motifs for the wavelength range above the 500 nm and a sharp positive slope higher than 560 nm in contrast to yellow motifs that present the sharp positive slope at lower wavelengths. On the other hand, the dominant wavelength (λ_D_) varies between 583 and 599 nm for red pigments, between 578 and 582 nm for yellow pigments and between 575 and 584 nm for unpainted rock supports. Therefore, we can observe that λ_D_ values differentiate the red and yellow pigments but theλ_D_ values for supports and yellow pigments are overlapped.

### EDXRF analysis

We recorded a total of 128 EDXRF spectra on red pigments and 22 EDXRF spectra on yellow pigments from the 67 selected plaquettes with red motifs and 14 selected plaquettes with yellow motifs (Tables 2 and 3), as well as 106 EDXRF spectra on unpainted zones of the plaquettes. In order to determine the composition of the rock support, powdered samples from unpainted rock substrates were analyzed by X-ray diffraction (XRD). These analyses reveal the presence of calcite, dolomite and quartz as main crystalline phases that are characteristic of the limestone-sandstone sedimentary rocks [16]. The elemental composition of these crystalline phases is reflected in the EDXRF spectra that show intense fluorescence lines of Ca, minor intense fluorescence lines of Fe, Mn, Ti, K and Si and, in some cases, traces of Sr and Zr. On the other hand, most of these elements are present in red and yellow iron based pigments as hematite, goethite and other iron oxides where Fe is the “key element” characterized by an intense fluorescence signal that is easy to detect and does not interfere with lines from other elements present in the pigments.

However, the presence of a common set of elements (with different concentrations) in the red and yellow painted zones and in the unpainted zones of the plaquettes, make it difficult to discriminate the elemental composition of the pigments from the rock surface background. Therefore, in order to distinguish between the elemental composition of the superficial pigment layers and that of the underlying rock, the recorded spectra from painted areas were compared with spectra from unpainted areas. On the basis that the EDXRF spectrum of a colored zone with an iron based pigment presents higher iron peaks than the unpainted rock surface of the plaquette, a discussion based on the Fe/Ca fluorescence peak ratios was made.

**Fig 5:**
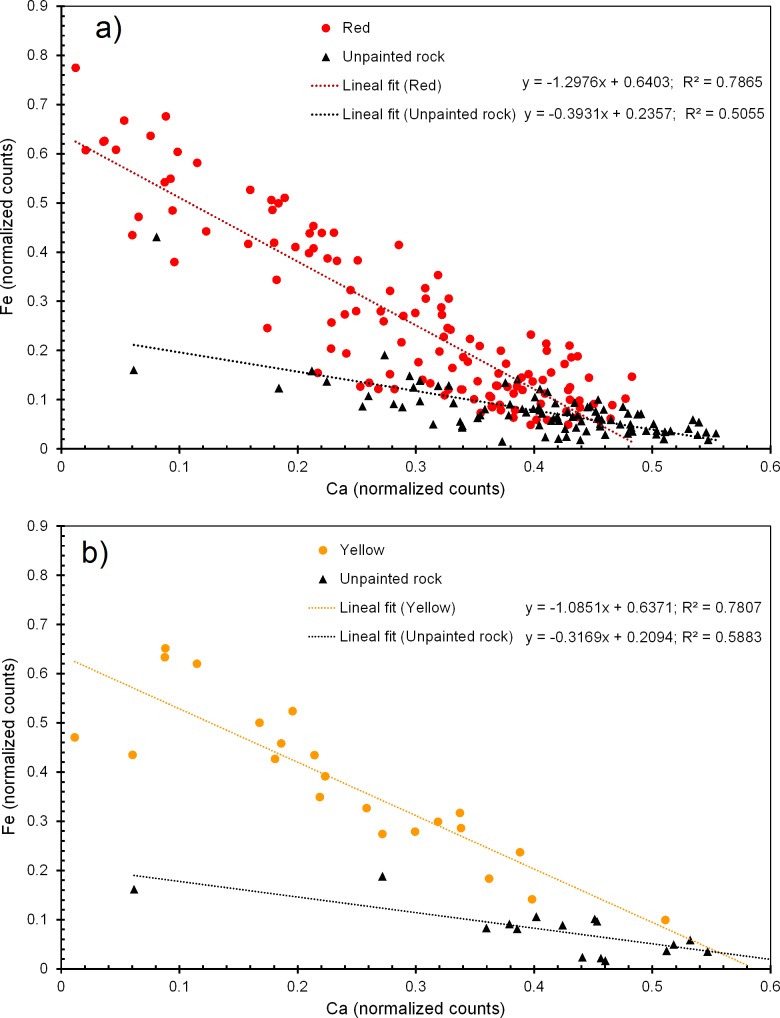
Normalized net areas of the Fe K-lines versus the normalized net areas of the Ca K-lines from the painted and unpainted zones of the Parpallo’s Cave plaquettes. (a) Motifs with red pigments. (b) Motifs with yellow pigments.

Fig 5 shows the scatter-plot of the normalized K-lines of iron and calcium from the analyzed points of the red and yellow motifs. In both cases, the intensity of the Fe peaks from colored zones is greater than in the nude rock and the linear tendency of the Fe versus Ca signals shows a negative slope with significant differences between painted and unpainted zones. The anti-correlation between Ca and Fe observed in the rock substrates is characteristic of the weathering processes as iron replaces calcium in the rock surface layers. The anti-correlation in the pictorial motifs will be associated with the presence of red and yellow iron based pigment layers on top of the calcareous rock substrates.

**Fig 6:**
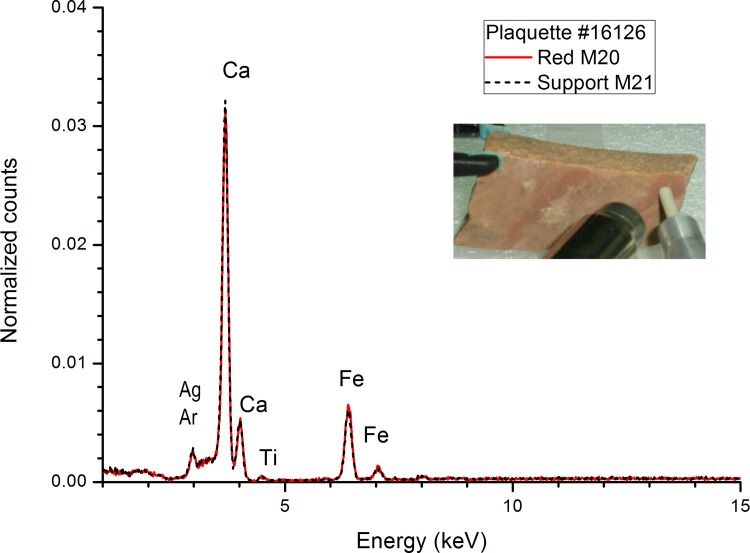
Plaquette 16126. **EDXRF spectra of the light red pigment (legs of a zoomorphic motif) and the rock support.** The fluorescence peaks at 3 keV (Ag) and 8 keV (Cu) come from the X-ray tube and the detector framework.

**Fig 7:**
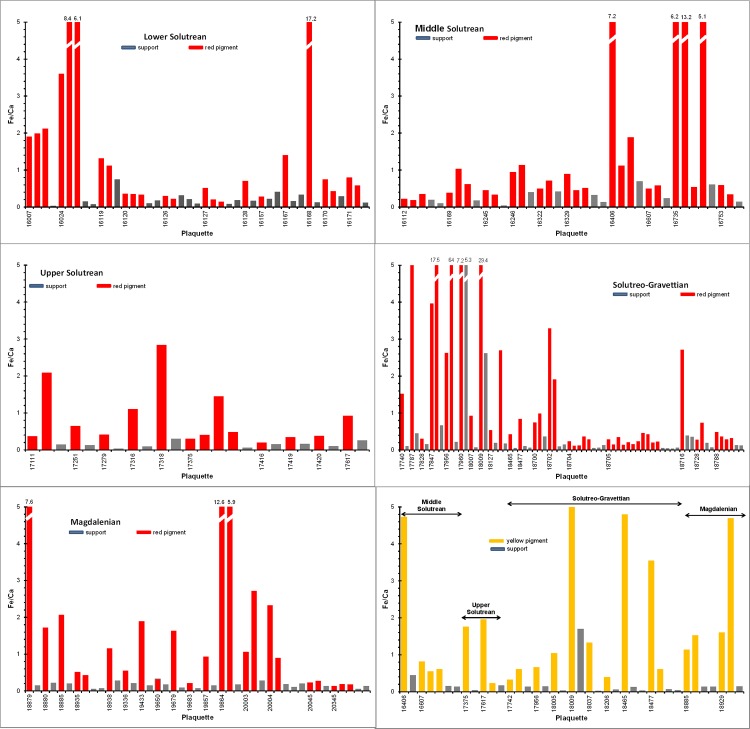
Fe/Ca ratios from the EDXRF spectra obtained in red and yellow motifs and rock supports.

Nevertheless, a number of EDXRF measurements from pigmented motifs present iron levels similar to the unpainted rock surfaces. In these cases, the EDXRF spectra of the pigmented zones are undistinguishable of the unpainted rock surfaces (Fig 6) and both spectra present similar low Fe/Ca ratios with intense signals of calcium and low signals of iron (see Fig 7 and S3 and S4 Tables). This fact would be due to the expected fluctuations of the fluorescence signals in thin or deteriorated pigment layers or in pigment layers highly absorbed into the rock support. Such is the case of the signs and zoomorphic motifs from the plaquettes 16126, 16112, 16322, 16753, 17960, 18704 (face A), 20345 (face B), the ramiform motif from the plaquette 20045, the red dot of the plaquette 16127 (face B) or the weak red line presents in the plaquette 17416.

**Fig 8:**
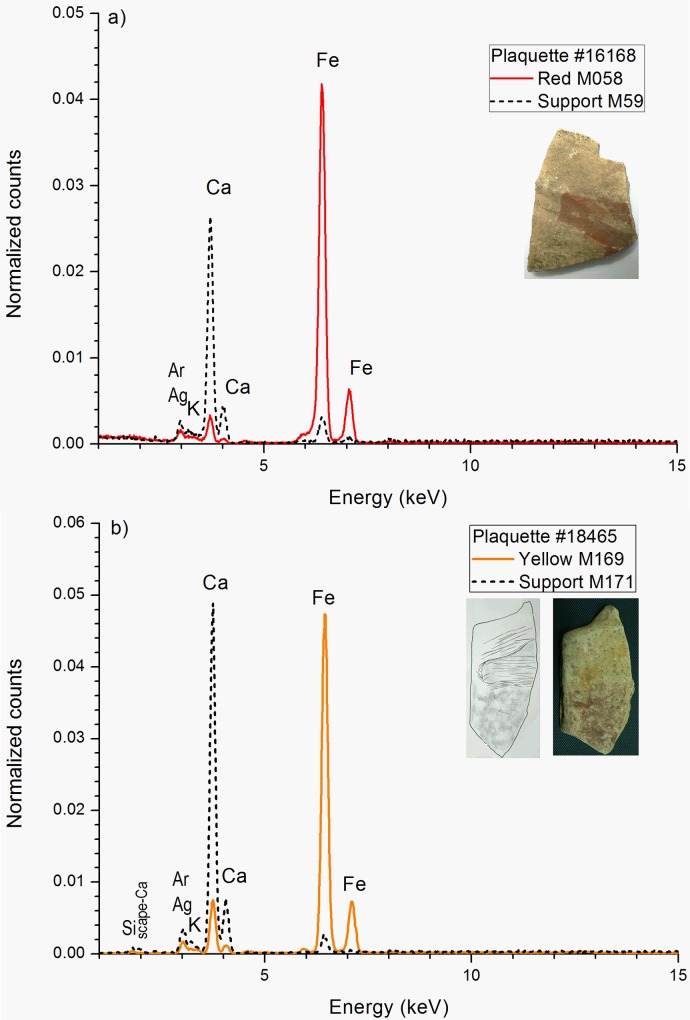
Comparison between EDXRF spectra of red and yellow iron based pigments and that of the rock support. (a) Red zoomorphic motif on plaquette 16168. (b) Yellow zoomorphic motif on plaquette 18465. (The fluorescence peaks at 3 keV (Ag) and 8 keV (Cu) come from the X-ray tube and the detector framework).

Conversely, most plaquettes with red motifs and all the plaquettes with yellow motifs present significant differences in Fe content when we compare the EDXRF spectra from the red or yellow motifs and the underlying rock surfaces. In these plaquettes the Fe/Ca ratios in the pictorial motifs are higher than in the bare rock (see Fig 7 and S3 and S4 Tables). These high iron levels are characteristic of well-defined motifs with prominent pigment layers and intense colorations, and confirm the presence of iron based mineral compounds. As representative examples, in Fig 8 we show the EDXRF spectra of the red snout of a zoomorphic motif recorded on the plaquette 16168 and the yellow horse head recorded on the plaquette 18465. In these cases, the intensity of the iron signal and the Fe/Ca ratio are more intense in the pigment layer than in the underlying rock and the calcium peaks of the rock support are attenuated by deep red or yellow pigments with a dense pictorial layer.

With respect to the raw material used as red or yellow pigments in the plaquettes of the chronological sequence included in this work, the EDXRF analyses only confirm the use of iron based pigments (oxides and/or hydroxides for red and yellow pigments) but these analyses cannot specify the type (molecular composition) of raw materials that were used as pigments. However, some red and yellow motifs of several plaquettes show different spectra for certain elements like manganese, arsenic or lead. Manganese was detected as component of the iron based red pigments in zoomorphic motifs of the plaquettes 18704 (face A), 18788 (face A) and the painted surface of the 20004 (face A). Arsenic was detected in the zoomorphic motif of the plaquette 16735. Lead was detected in the iron based yellow pigment on the painted surface of the plaquette 18929. In these plaquettes, the net areas of the manganese, arsenic or lead fluorescence lines of the pigments are significantly higher than in the rock supports, whereas for the rest of plaquettes these elements have not been detected or the net areas of these elements in the painted motifs and the rock supports are similar (S3 and S4 Tables). The presence of Mn, As and Pb could be associated to pigment raw materials from different ores [27]. In relation to the elemental composition of the rock supports, strontium and zirconium are discriminatory elements in a certain number of plaquettes (S3 and S4 Tables) that indicate differences in the geogenesis of the rock supports included in this study. As conclusion, we can consider that the artists used specific raw materials with different geological fingerprints characterized by the presence or absence of few elements (manganese, arsenic or lead into the red/yellow iron oxide matrix; strontium and zirconium into the rock support).

Pictorial motifs that are recognizable as anthropomorphous, zoomorphous, or well defined geometric figures would be catalogued as artistic or symbolic representations and we can postulate the anthropic origin of the decoration. However, if the pictorial motifs are vague forms and these forms are difficult to catalog or interpret, we cannot exclude that the pigmented areas are the result of a surface patination of the plaquettes associated with geogenic, biogenic, weathering, firing or post-depositional processes in the archaeological site. In any case and if sampling is allowed, additional microscopic, elemental, molecular and geological analysis should be performed to complement the EDXRF analyses and provide a holistic information about the nature and composition of the red and yellow pigmented areas of the plaquettes.

### FT-IR analysis

Due to the difficulty to identify the composition of the inorganic pigments present in red and yellow motifs by EDXRF, we used FT-IR spectrometry as a complementary analytical tool that can help us in a preliminary identification of the main inorganic molecular components present in pigment layers [28].

In order to fulfill the restrictions of regional heritage politics of preservation, FT-IR analyses were performed only on detached microsamples from a limited set of plaquettes with red and yellow motifs (Table 4). This selection was only based on the visual perception to obtain the microsamples in an easy mode avoiding an aggressive sampling. Samples collected, in a previous work [16], on the unpainted rock surface of the plaquettes revealed evidence of infrared bands of calcite, quartz, dolomite and clays, witch match with the elemental composition detected by EDXRF [16]. Red and yellow pigment samples correspond to detached grains that were collected from pigment layers bound to the rock beneath. Thus, it was not possible to separate them completely from the substrate, and therefore these microsamples can contain mineralogical components from the pigment layers and rock substrates.

**Table 4:** FT-IR analyses of the selected plaquettes with red and yellow colored motifs.

Plaquette	Chronology	Pigment	Description	Identified compounds and most intense FT-IR bands (cm^-1^)
**16119 (face B)**	**LS**	**Red**	**Extended paint**	**Hematite (532, 464) ; Calcite (1450, 876, 1799, 2514); Quartz (1032); Kaolinite (3700, 3621, 913)**
**16168**	**LS**	**Red**	**Undefined zoomorph: snout**	**Hematite (540, 450); Quartz (1038); Calcite (1436)**
**17375 (face A)**	**US**	**Red**	**Extended paint**	**Calcite (1441, 874, 1796, 2521); Quartz (1030)**
**17740**	**SGI**	**Red**	**Extended paint**	**Hematite (542, 476); Calcite (1434, 878, 1818, 2524); Quartz (1035)**
**18009 (face A)**	**SGI**	**Red**	**Extended paint**	**Hematite (558, 469); Calcite (1442, 880); Quartz (1032)**
**18465**	**SGIII**	**Red**	**Undefined: extended stain**	**Calcite (1442, 876, 1800, 2518); Quartz (1035)**
**18716**	**SGIII**	**Red**	**Undefined zoomorfic motif**	**Hematite (540, 469); Calcite (1442); Quartz (1035); Kaolinite (3698, 3628)**
**18879**	**AMa**	**Red**	**Extended paint**	**Quartz (1040); Calcite (1440); Kaolinite (3700, 3620)**
**19864**	**UM**	**Red**	**Undefined: extended stain**	**Hematite (522, 474), Calcite (1479, 1809, 2525, 879); Quartz (1030)**
**16607 (face A)**	**RMS**	**Yellow**	**Undefined sign**	**Goethite (3148, 905, 798); Calcite (1466); Quartz (1032)**
**17375 (face A)**	**US**	**Yellow**	**Undefined sign**	**Goethite (3133, 908, 801); Calcite 1434, 876, 1796, 2516); Quartz (1031)**
**18009 (face A)**	**SG-I**	**Yellow**	**Undefined sign**	**Goethite (3160, 912, 801); Quartz (1080); Kaolinite (3698, 3620)**
**18206**	**SG-II**	**Yellow**	**Undefined sign**	**Goethite (3152, 900, 798); Calcite (1457, 1797, 2515); Quartz (1035); Kaolinite (3698, 3621, 1000)**
**18465**	**SG-III**	**Yellow**	**Undefined zoomorfic motif**	**Goethite (3146, 892, 795); Quartz (1034); Calcite (1442, 1797, 2524)**
**18477**	**SG-III**	**Yellow**	**Undefined zoomorfic motif**	**Goethite (3144, 892, 801); Calcite (1410); Quartz (1030)**

**Fig 9:**
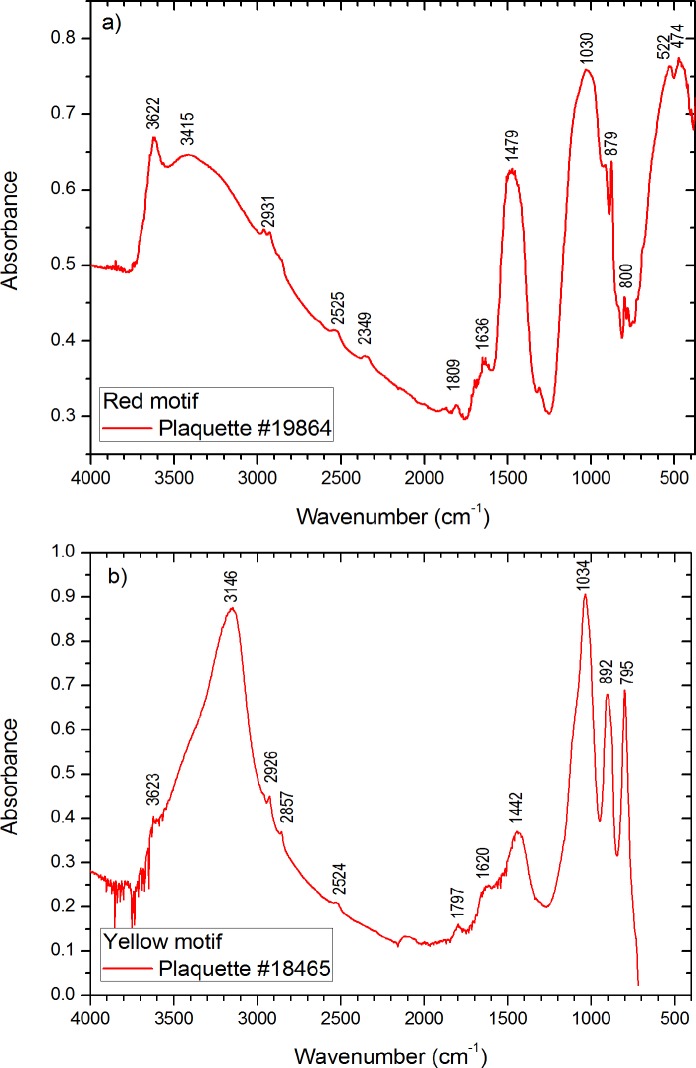
Examples of FTIR spectra. (a) Red motif of the plaquette 19864. (b) Yellow motif of the plaquette 18465.

FT-IR analyses of red pigments indicate the presence of hematite and other minerals in six out of nine samples (see Table 4). Hematite is identified by the presence of the characteristic bands at the intervals of 560–520 and 480–440 cm^-1^. As example, Fig 9A shows the FT-IR spectrum of the red motif from the plaquette 19864 with the stretching and torsional modes of hematite Fe-O bonds at 522 and 474 cm^-1^, and other main components as quartz (1100–1000 cm^-1^), calcite (1479, 879, 1809, 2525 cm^-1^) and the presence of bands in the higher wavenumber region attributed to OH and H_2_O stretching vibrations (3622, 3415 cm^-1^) and bending vibrations of OH bonds (1636 cm^-1^).

The analysis of the inorganic fraction of pigment samples from the Parpalló plaquettes by FT-IR indicates the presence of iron hydroxides as goethite in all of the analyzed yellow samples. Yellow samples also present accessory compounds often associated with earth minerals as quartz, carbonates and clays. Fig 9B (plaquette 18465) is representative of nearly all the FT-IR spectra from yellow pigments and shows the characteristic goethite bands with a strong hydroxyl stretch at about 3146 cm^-1^, two hydroxyl bending bands around 892 and 795 cm^-1^, respectively, and exhibit a peak between 1100 and 1000 cm^-1^ because of the Si-O-Si stretchings. Calcite is identified in this spectrum at 1442, 1797 and 2524 cm^-1^.

The position and intensities of these bands are for guidance only because can vary with particle size and shape of the samples and can be also affected by the presence of accessory minerals [22, 26]. Identified compounds in the analyzed plaquettes are shown in Table 4. These analyses suggest that pigmented areas were the result of the application of red and yellow iron oxides that in their natural forms incorporate other compounds as calcium carbonates, clays and quartz in their natural forms that a priori we cannot interpret as loading agents (extenders or fillers). On the other hand, FT-IR absorption bands of organic compounds were not observed in both red and yellow motifs.

## Conclusions

The plaquettes from the Parpalló Cave constitutes one of the most important collections of Palaeolithic portable art. One of the peculiarities of the collection is the high number of plaquettes with zoomorphic and sign depictions painted with red and yellow pigments, particularly in relation to Solutrean chronology. Given the cultural and archaeological significance of this collection, the characterization of the pigments is of great interest for archaeology, history and art conservation in the context of the Palaeolithic rock art. On the other hand, this characterization opens the possibility to establish comparative studies with the parietal art of the Iberian Mediterranean region.

The combination of analytical techniques as spectrophotometry in the visible region, energy dispersive X-ray fluorescence spectrometry and infra-red spectroscopy have provide information about the chromatic coordinates and chemical composition of the red and yellow pigments of the pictorial motifs present in the Parpalló plaquettes stored at the Museo de Prehistoria of Valencia.

Color measurements by means of in situ spectrophotometry were recorded on the pictorial motifs with the aim of displaying similitudes/differences between the red and yellow pigments. The obtained data show significant differences between the rock supports and the pictorial motifs. The acquired colorimetric coordinates and the spectral curves will provide a useful reference for further studies as the fading of the pigments and the variation of the chromatic coordinates with time.

In this study, an in situ and non-destructive EDXRF qualitative comparative analysis was performed on the red and yellow motifs and the non-pigmented surfaces of the plaquettes using the same methodological approach (instrumental and settings). From EDXRF measurements, several conclusions were drawn concerning the elemental composition of the red and yellow pigments. Iron is the key element of the whole of the analyzed pigments but no differentiation among different iron oxides can be established. When we compare the spectra between colored motifs and the rock supports, the Fe/Ca fluorescence peak ratios from the most of the colored motifs are higher than the ratios from the nude rocks. On the contrary, several colored motifs present indistinguishable spectra and then similar Fe/Ca ratios in the painted zone and in the rock supports. If these motifs are zoomorphic or well defined geometric representations (signs) there is no doubt about their deliberate anthropogenic origin and the observed undistinguishable spectra and similar Fe/Ca ratios could be associated with a deep penetration of the pigment layer into de rock support or with a thin or deteriorated (weathered) pictorial layer. In any case, further investigations on sampled pigments from these motifs would be accomplished to analyze and characterize them by means of other analytical techniques that require sampling. Only in that way can we distinguish between anthropic and non-anthropic depictions.

Additionally, as far as qualitative EDXRF analysis is concerned, we have identified the same elemental composition in the red and yellow pictorial motifs from the Gravetian, Solutrean and Magdalenian levels suggesting that the pigment sources would be mainly related to the availability of raw materials within the surrounding areas of the Parpalló Cave. The detection of manganese, arsenic or lead only on a small set of plaquettes constitutes a chemical signature of these red and yellow pigments which may indicate specific raw material sources.

FT-IR analyses did facilitate the identification of hematite in red motifs, goethite in yellow motifs and unspecific iron oxides. These ochreous compounds were detected in combination with other compounds as calcium carbonates, quartz and clay minerals whose presence is common in red and yellow earths. Calcium carbonates, quartz and clays have also been detected in the rock substrates. The recorded FT-IR spectra do not match with the presence of organic binders.

One limitation of this work is the restriction, imposed by the authorities responsible for cultural heritage conservation, to carry out in situ and non-destructive analyses. Spectrophotometry and EDXRF spectrometry have been applied in situ and in a non-destructive mode at the Museo de Prehistoria de Valencia. Only detached grains from certain plaquettes were analyzed by FT-IR in the laboratory. Therefore, more thorough analyses of the red and yellow motifs, present in the plaquettes of the Parpalló cave, using complementary physicochemical techniques, need to be undertaken to completely characterize this unique, exceptional and valuable Palaeolithic portable art.

## Supporting Information

S1 Table Summary of the analyzed plaquettes from the Parpalló cave with red motifs. (DOCX)

S2 Table Summary of the analyzed plaquettes from the Parpalló cave with yellow motifs. (DOCX)

S3 Table Normalized net areas of the elements detected by EDXRF in red motifs (r) and rock supports (s) from the Parpalló plaquettes. (n.d. = non detected).
(DOCX)

S4 Table Normalized net areas of the elements detected by EDXRF in yellow (y) motifs and rock supports (s) from the Parpalló plaquettes. (n.d. = non detected).
(DOCX)
