# Supplementary material for: A Unique Collection of Palaeolithic Painted Portable Art: Characterization of Red and Yellow Pigments from the Parpalló Cave (Spain)
Source: PLoS One. 2016 Oct 12;11(10):e0163565. doi: 10.1371/journal.pone.0163565 (PMC5061316; doi:10.1371/journal.pone.0163565)
Supplement: S1 Table — (DOCX) [file pone.0163565.s001.docx]

S1_ Supporting_Information_Table. **Summary of the analyzed plaquettes from the Parpalló cave with red motifs.**

| **Plaquette**  **identification number** | **Chronology** | **Size (mm) (width, height, thickness)** | **Description** |
| --- | --- | --- | --- |
| **16007** | **LS** | **104, 71, 14** | **Painted surface** |
| **16119 (face B)** | **LS** | **450, 350, 44** | **Sign: Rectangle** |
| **16120 (face A)** | **LS** | **450, 350, 44** | **Zoomorph: auroch** |
| **16024** | **LS** | **112, 76, 27** | **Probably part of zoomorph** |
| **16126** | **LS** | **63, 32, 11** | **Zoomorph: legs** |
| **16127 (face A)** | **LS** | **52, 53, 11** | **Painted surface** |
| **16127 (face B)** | **LS** | **52, 53, 11** | **Sign : points** |
| **16128** | **LS** | **100, 63, 7** | **Sign: probably rectangle and lines** |
| **16157** | **LS** | **100, 77, 7** | **Zoomorph: legs** |
| **16167** | **LS** | **210, 101, 32** | **Sign: parallel lines** |
| **16168** | **LS** | **142, 106, 26** | **Zoomorph: snout** |
| **16170** | **LS** | \| **151,116,12** \| \| --- \| | **Sign : line** |
| **16171** | **LS** | **155,89,11** | **Part of probable zoomorph** |
| **16112** | **LS-AMS** | **130,110,29** | **Zoomorph: incomplete** |
| **16169** | **AMS** | **113,108,21** | **Zoomorph: ibex** |
| **16245** | **AMS** | **112,95,32** | **Painted surface** |
| **16246** | **AMS** | **97,68,11** | **Painted surface** |
| **16322** | **AMS** | **73,52,11** | **Zoomorph: legs** |
| **16329** | **AMS** | **118,52,11** | **Zoomorph: legs** |
| **16406 (face A)** | **AMS** | **123,45,12** | **Sign : indetermined (bichrome)** |
| **16406 (face B)** | **AMS** | **123,45,12** | **Sign : indetermined (bichrome)** |
| **16607 (face A)** | **RMS** | **160,144,27** | **Painted surface** |
| **16735** | **RMS** | **172,150,18** | **Zoomorph: female cervid** |
| **16753** | **RMS** | **60,45,19** | **Sign: oval** |
| **17111** | **US** | **41,30,2** | **Zoomorph: indeterminate** |
| **17251** | **US** | **82,50,18** | **Painted surface (limited)** |
| **17279** | **US** | **70,42,15** | **Painted surafce (bichrome)** |
| **17316** | **US** | **100,76,31** | **Painted surface (limited)** |
| **17318** | **US** | **67,49,13** | **Probably incomplete zoomorph** |
| **17375 (face A)** | **US** | **83, 45, 13** | **Painted surface (bichrome)** |
| **17375 (face B)** | **US** | **83, 45, 13** | **Painted surface** |
| **17416** | **US** | **93,65,7** | **Sign: wide line?** |
| **17419** | **US** | **83,48,23** | **Sign: lines in succession** |
| **17420** | **US** | **92,29,20** | **Sign: rectangle** |
| **17617 (face B)** | **US** | **65,55,15** | **Painted bichrome surface** |
| **17740** | **SGI** | **48,26,11** | **Painted surface** |
| **17787** | **SGI** | **66,56,30** | **Sign: curved line** |
| **17828** | **SGI** | **93,17,13** | **Sign: point** |
| **17847** | **SGI** | **102,73,10** | **Painted surface** |
| **17956 (face A)** | **SGI** | **62, 51, 11** | **Painted bichrome surface** |
| **17956 (face B)** | **SGI** | **62, 51, 11** | **Painted surface** |
| **17960** | **SGI** | **76, 60, 15** | **Sign: limited painted surface** |
| **18007** | **SGI** | **86, 84, 19** | **Painted surface** |
| **18009 (face A)** | **SGI** | **76, 62, 13** | **Painted surface** |
| **18127** | **SGII** | **151, 62, 17** | **Zoomorph: snout horse** |
| **18227** | **SGII** | **128, 90, 16** | **Sign? Limited painted surface** |
| **18700** | **SGII-SGIII** | **138, 74, 16** | **Probably incomplete zoomorph** |
| **18702** | **SGII-SGIII** | **91, 80, 15** | **Zoomorph: indeterminate** |
| **18465** | **SGIII** | **193, 52, 15** | **Painted surface, delimiting horse head (bichrome)** |
| **18477** | **SGIII** | **61, 35, 6** | **Zoomorph: indetermiate (yellow) and exterior painted surface (red)** |
| **18704 (face A)** | **SGIII** | **218, 115, 23** | **Zoomorph: ibex** |
| **18705** | **SGIII** | **375, 271, 45** | **Zoomorph: horse** |
| **18716** | **SGIII** | **81, 24, 16** | **Painted surface** |
| **18728 (face A)** | **SGIII** | **80, 66, 12** | **Zoomorph: cervid antlers** |
| **18788 (face A)** | **SGIII** | **333, 221,27** | **Sign: triangles** |
| **18788 (face B)** | **SGIII** | **333, 221, 27** | **Zoomorph: indeterminate** |
| **18879** | **AMa** | **79, 68, 11** | **Painted surface** |
| **18880** | **AMa** | **41, 41, 6** | **Sign or zoomorph snout** |
| **18885 (face B)** | **AMa** | **54, 40, 9** | **Painted bichrome surface** |
| **18935 (face A)** | **AMa** | **76, 50, 14** | **Painted surface** |
| **18938 (face B)** | **AMa** | **68, 44, 6** | **Painted surface (limited)** |
| **19336** | **AMb** | **89, 65, 18** | **Painted surface** |
| **19433** | **AMb** | **98, 52, 11** | **Painted surface** |
| **19650 (face B)** | **AMb** | **89, 66, 6** | **Probably incomplete zoomorph** |
| **19679 (face A)** | **AMb** | **67, 57, 13** | **Painted surface** |
| **19683 (face B)** | **AMb** | **74, 62, 8** | **Painted surface** |
| **19864** | **UM** | **190, 132, 35** | **Painted surface** |
| **19857 (face B)** | **UM** | **55, 45, 14** | **Painted surface (limited)** |
| **20003** | **UM** | **130, 130, 40** | **Painted surface** |
| **20004 (face A)** | **UM** | **150, 120, 30** | **Painted surface** |
| **20004 (face B)** | **UM** | **150, 120, 30** | **Painted surface** |
| **20045** | **UM** | **109, 66, 26** | **Sign: ramiform** |
| **20345 (face A)** | **UM** | **112, 107, 7** | **Zoomorph: ibex** |
| **20345 (face B)** | **UM** | **112, 107, 7** | **Zoomorph: indeterminate** |

**LS: Lower Solutrean (21000-20500 BP). AMS and SMS: Ancient and Recent Middle Solutrean (20500-20000 BP). US: Upper Solutrean (2000-19500 BP). SG-I, SGII and SGIII: Solutreo-Gravettian I, II and III (19500-17000 BP). AM: Ancient Magdalenian (17000-145000 BP). UM: Midlde and Upper Magdalenian (14.500-12.000). Chronologies are only indicatives.**
