# Supplementary material for: A Unique Collection of Palaeolithic Painted Portable Art: Characterization of Red and Yellow Pigments from the Parpalló Cave (Spain)
Source: PLoS One. 2016 Oct 12;11(10):e0163565. doi: 10.1371/journal.pone.0163565 (PMC5061316; doi:10.1371/journal.pone.0163565)
Supplement: S2 Table — (DOCX) [file pone.0163565.s002.docx]

S2_ Supporting_Information_Table. **Summary of the analyzed plaquettes from the Parpalló cave with yellow motifs.**

| **Plaquette**  **identification number** | **Chronology** | **Size (mm) (width, height, thickness)** | **Description** |
| --- | --- | --- | --- |
| **16406 (face A)** | **AMS** | **123,45,12** | **Painted surface (bichrome)** |
| **16607 (face A)** | **RMS** | **160,144,27** | **Painted surface (limited)** |
| **16607 (face B)** | **RMS** | **160,144,27** | **Painted surface (limited)** |
| **17375 (face A)** | **US** | **83,45,13** | **Painted surface (bichrome)** |
| **17617 (face B)** | **US** | **65,55,15** | **Painted surface (bichrome)** |
| **17742 (face A)** | **SG-I** | **50,37,9** | **Sign: lines** |
| **17956 (face A)** | **SG-I** | **62,51,11** | **Painted surface (bichrome)** |
| **18005** | **SG-I** | **75,70,17** | **Painted surface** |
| **18009 (face B)** | **SG-I** | **76,62,13** | **Painted surface** |
| **18037 (face B)** | **SG-II** | **107,88,18** | **Painted surface** |
| **18206** | **SG-II** | **85,70,15** | **Painted surface** |
| **18465** | **SG-III** | **193,52,15** | **Zoomorph: Horse’s snout (bichrome)** |
| **18477** | **SG-III** | **61,35,6** | **Zoomorph: indetermiate (yellow) and exterior painted surface (red)** |
| **18885 (face A)** | **AM** | **54,40,9** | **Painted surface (bichrome)** |
| **18885 (face B)** | **AM** | **54,40,9** | **Painted surface (limited)** |
| **18929** | **AM** | **46,34,10** | **Painted surface (limited)** |

**LS: Lower Solutrean (21000-20500 BP). AMS and RMS: Ancient and Recent Middle Solutrean (20500-20000 BP). . US: Upper Solutrean (2000-19500 BP). SG-I, SGII and SGIII: Solutreo-Gravettian I, II and III (19500-17000 BP). AM: Ancient Magdalenian (17000-145000 BP). UM: Midlde and Upper Magdalenian (14.500-12.000). Chronologies are only indicatives.**
