# Supplementary material for: A Unique Collection of Palaeolithic Painted Portable Art: Characterization of Red and Yellow Pigments from the Parpalló Cave (Spain)
Source: PLoS One. 2016 Oct 12;11(10):e0163565. doi: 10.1371/journal.pone.0163565 (PMC5061316; doi:10.1371/journal.pone.0163565)
Supplement: S3 Table — (n.d. = non detected). (DOCX) [file pone.0163565.s003.docx]

S3_ Supporting_Information_Table. Normalized net areas of the elements detected by EDXRF in red (r) motifs and rock supports (s) from the Parpalló plaquettes. (n.d.= non detected).

| Plaquette # | Face | Code | Period | Color | **Si** | **K** | **Ca** | **Ti** | **Mn** | **Fe** | **As** | **Sr** | **Zr** |
| --- | --- | --- | --- | --- | --- | --- | --- | --- | --- | --- | --- | --- | --- |
| 16007 |  | **M006** | LS | **r** | 0.00174 | 0.00404 | 0.23060 | 0.00076 | 0.00197 | 0.43955 | n.d. | 0.00352 | n.d. |
| 16007 |  | **M007** | LS | **r** | 0.00132 | 0.00597 | 0.22037 | 0.00127 | 0.00202 | 0.43902 | n.d. | 0.00441 | n.d. |
| 16007 |  | **M008** | LS | **r** | 0.00209 | 0.00648 | 0.21331 | 0.00124 | 0.00165 | 0.45303 | n.d. | 0.00456 | n.d. |
| 16007 |  | **M009** | LS | **s** | 0.00334 | 0.00372 | 0.54725 | 0.00050 | 0.00178 | 0.01866 | n.d. | 0.00718 | n.d. |
| 16024 |  | **M001** | LS | **r** | 0.00306 | 0.01009 | 0.12264 | 0.00699 | 0.00717 | 0.44215 | n.d. | n.d. | n.d. |
| 16024 |  | **M002** | LS | **r** | 0.00197 | 0.00484 | 0.07559 | 0.00310 | 0.00865 | 0.63656 | n.d. | n.d. | n.d. |
| 16024 |  | **M003** | LS | **r** | 0.00177 | 0.00369 | 0.09840 | 0.00292 | 0.00550 | 0.60379 | n.d. | n.d. | n.d. |
| 16024 |  | **M004** | LS | **s** | 0.00601 | 0.00783 | 0.43704 | 0.00431 | 0.00148 | 0.06856 | n.d. | n.d. | n.d. |
| 16024 |  | **M005** | LS | **s** | 0.00584 | 0.00522 | 0.50377 | 0.00284 | 0.00155 | 0.03718 | n.d. | n.d. | n.d. |
| 16119 | B | **M034** | LS | **r** | 0.00848 | 0.00317 | 0.24468 | 0.00258 | 0.00404 | 0.32268 | n.d. | n.d. | n.d. |
| 16119 | B | **M035** | LS | **r** | 0.01134 | 0.00217 | 0.24941 | 0.00283 | 0.00829 | 0.28013 | n.d. | n.d. | n.d. |
| 16119 | B | **M036** | LS | **s** | 0.01141 | 0.00725 | 0.21209 | 0.00595 | 0.00416 | 0.15881 | n.d. | n.d. | n.d. |
| 16120 | A | **M029** | LS | **r** | 0.00553 | 0.01395 | 0.33825 | 0.00464 | 0.00091 | 0.12083 | n.d. | n.d. | n.d. |
| 16120 | A | **M030** | LS | **r** | 0.00597 | 0.01575 | 0.33927 | 0.00411 | 0.00035 | 0.12046 | n.d. | n.d. | n.d. |
| 16120 | A | **M032** | LS | **r** | 0.01573 | 0.00555 | 0.32420 | 0.00341 | 0.00488 | 0.10902 | n.d. | n.d. | n.d. |
| 16120 | A | **M031** | LS | **s** | 0.00490 | 0.00701 | 0.43556 | 0.00170 | 0.00051 | 0.04370 | n.d. | n.d. | n.d. |
| 16120 | A | **M033** | LS | **s** | 0.01670 | 0.00378 | 0.35232 | 0.00277 | 0.00363 | 0.06316 | n.d. | n.d. | n.d. |
| 16126 |  | **M018** | LS | **r** | 0.00662 | 0.01153 | 0.36221 | 0.00469 | 0.00118 | 0.10790 | n.d. | n.d. | n.d. |
| 16126 |  | **M020** | LS | **r** | 0.00510 | 0.01172 | 0.40565 | 0.00436 | 0.00170 | 0.09168 | n.d. | n.d. | n.d. |
| 16126 |  | **M019** | LS | **s** | 0.00666 | 0.01421 | 0.30347 | 0.00524 | 0.00122 | 0.09739 | n.d. | n.d. | n.d. |
| 16126 |  | **M021** | LS | **s** | 0.00489 | 0.01303 | 0.40401 | 0.00440 | 0.00080 | 0.08555 | n.d. | n.d. | n.d. |
| 16126 |  | **M022** | LS | **s** | 0.00518 | 0.00999 | 0.42619 | 0.00206 | 0.00412 | 0.04105 | n.d. | n.d. | n.d. |
| 16127 | A | **M040** | LS | **r** | 0.00385 | 0.00532 | 0.34385 | 0.00609 | 0.00718 | 0.17736 | n.d. | n.d. | n.d. |
| 16127 | B | **M037** | LS | **r** | 0.00697 | 0.00326 | 0.35468 | 0.00496 | 0.00563 | 0.07307 | n.d. | n.d. | n.d. |
| 16127 | B | **M038** | LS | **r** | 0.00663 | 0.00275 | 0.40199 | 0.00494 | 0.00348 | 0.05925 | n.d. | n.d. | n.d. |
| 16127 | A | **M041** | LS | **s** | 0.00482 | 0.00016 | 0.44434 | 0.00253 | 0.00302 | 0.03635 | n.d. | n.d. | n.d. |
| 16127 | B | **M039** | LS | **s** | 0.00704 | 0.00313 | 0.35449 | 0.00594 | 0.01160 | 0.06843 | n.d. | n.d. | n.d. |
| 16128 |  | **M042** | LS | **r** | 0.00382 | 0.00728 | 0.32350 | 0.00509 | 0.00741 | 0.22788 | n.d. | n.d. | n.d. |
| 16128 |  | **M043** | LS | **s** | 0.00440 | 0.00449 | 0.42828 | 0.00413 | 0.01125 | 0.07579 | n.d. | n.d. | n.d. |
| 16157 |  | **M049** | LS | **r** | 0.00409 | 0.01222 | 0.34913 | 0.00571 | 0.01918 | 0.10092 | n.d. | n.d. | n.d. |
| 16157 |  | **M051** | LS | **s** | 0.00658 | 0.01333 | 0.35835 | 0.00644 | 0.00251 | 0.08085 | n.d. | n.d. | n.d. |
| 16157 |  | **M053** | LS | **s** | 0.01086 | 0.00899 | 0.25995 | 0.00826 | 0.00482 | 0.10791 | n.d. | n.d. | n.d. |
| 16167 |  | **M071** | LS | **r** | 0.01097 | 0.00544 | 0.17430 | 0.00418 | 0.01621 | 0.24549 | n.d. | n.d. | 0.00360 |
| 16167 |  | **M072** | LS | **s** | 0.01533 | 0.00142 | 0.31459 | 0.00155 | 0.00230 | 0.05013 | n.d. | n.d. | 0.00755 |
| 16167 |  | **M073** | LS | **s** | 0.00640 | 0.01132 | 0.25483 | 0.00587 | 0.00789 | 0.08649 | n.d. | n.d. | 0.00885 |
| 16168 |  | **M058** | LS | **r** | 0.00248 | 0.00502 | 0.03650 | 0.00396 | 0.01021 | 0.62618 | n.d. | n.d. | n.d. |
| 16168 |  | **M059** | LS | **s** | 0.01208 | 0.00364 | 0.33928 | 0.00383 | 0.00371 | 0.04349 | n.d. | n.d. | n.d. |
| 16170 | A | **M060** | LS | **r** | 0.01226 | 0.00857 | 0.28774 | 0.00421 | 0.00684 | 0.21661 | n.d. | n.d. | n.d. |
| 16170 | A | **M061** | LS | **r** | 0.01293 | 0.00524 | 0.28193 | 0.00483 | 0.00439 | 0.12156 | n.d. | n.d. | n.d. |
| 16170 | A | **M062** | LS | **s** | 0.01578 | 0.00314 | 0.28861 | 0.00400 | 0.00406 | 0.08533 | n.d. | n.d. | n.d. |
| 16171 | A | **M063** | LS | **r** | 0.00916 | 0.00784 | 0.24119 | 0.00569 | 0.00584 | 0.19412 | n.d. | n.d. | n.d. |
| 16171 | A | **M064** | LS | **r** | 0.00966 | 0.00825 | 0.30217 | 0.00573 | 0.01374 | 0.17627 | n.d. | n.d. | n.d. |
| 16171 | A | **M067** | LS | **s** | 0.00951 | 0.00809 | 0.40443 | 0.00246 | 0.00222 | 0.04863 | n.d. | n.d. | n.d. |
| 16112 |  | **M044** | LS-AMS | **r** | 0.00453 | 0.00318 | 0.43827 | 0.00401 | 0.00289 | 0.09836 | n.d. | n.d. | n.d. |
| 16112 |  | **M046** | LS-AMS | **r** | 0.00429 | 0.00758 | 0.46611 | 0.00287 | 0.00211 | 0.08895 | n.d. | n.d. | n.d. |
| 16112 |  | **M048** | LS-AMS | **r** | 0.00478 | 0.01300 | 0.36787 | 0.00630 | 0.00252 | 0.12852 | n.d. | n.d. | n.d. |
| 16112 |  | **M045** | LS-AMS | **s** | 0.00532 | 0.00735 | 0.40477 | 0.00587 | 0.00409 | 0.07882 | n.d. | n.d. | n.d. |
| 16112 |  | **M047** | LS-AMS | **s** | 0.00424 | 0.00591 | 0.50104 | 0.00252 | 0.00112 | 0.05108 | n.d. | n.d. | n.d. |
| 16169 |  | **M054** | AMS | **r** | 0.00698 | 0.00996 | 0.35211 | 0.00369 | 0.00301 | 0.13742 | n.d. | n.d. | n.d. |
| 16169 |  | **M055** | AMS | **r** | 0.00462 | 0.01044 | 0.27015 | 0.00361 | 0.00358 | 0.27988 | n.d. | n.d. | n.d. |
| 16169 |  | **M056** | AMS | **r** | 0.00525 | 0.01179 | 0.31990 | 0.00359 | 0.00585 | 0.19794 | n.d. | n.d. | n.d. |
| 16169 |  | **M057** | AMS | **s** | 0.00565 | 0.01096 | 0.37857 | 0.00447 | 0.00267 | 0.06851 | n.d. | n.d. | n.d. |
| 16245 |  | **M077** | AMS | **r** | 0.00994 | 0.00638 | 0.37620 | 0.00215 | 0.00109 | 0.17299 | n.d. | 0.00547 | n.d. |
| 16245 |  | **M079** | AMS | **r** | 0.01073 | 0.00875 | 0.37751 | 0.00302 | 0.00117 | 0.12608 | n.d. | 0.00431 | n.d. |
| 16245 |  | **M078** | AMS | **s** | 0.00971 | 0.00463 | 0.37305 | 0.00070 | 0.00148 | 0.01538 | n.d. | 0.00345 | n.d. |
| 16246 |  | **M074** | AMS | **r** | 0.00566 | 0.01145 | 0.27272 | 0.00404 | 0.00152 | 0.25936 | n.d. | n.d. | n.d. |
| 16246 |  | **M076** | AMS | **r** | 0.00875 | 0.00565 | 0.23976 | 0.00329 | 0.00712 | 0.27354 | n.d. | n.d. | n.d. |
| 16246 |  | **M075** | AMS | **s** | 0.00933 | 0.01021 | 0.31887 | 0.00585 | 0.01218 | 0.12824 | n.d. | n.d. | n.d. |
| 16322 |  | **M117** | AMS | **r** | 0.01617 | 0.00527 | 0.25291 | 0.00370 | 0.01303 | 0.12678 | n.d. | n.d. | n.d. |
| 16322 |  | **M118** | AMS | **r** | 0.01477 | 0.00536 | 0.21696 | 0.00537 | 0.01187 | 0.15458 | n.d. | n.d. | n.d. |
| 16322 |  | **M119** | AMS | **s** | 0.01626 | 0.00604 | 0.29806 | 0.00493 | 0.00711 | 0.12524 | n.d. | n.d. | n.d. |
| 16329 |  | **M120** | AMS | **r** | 0.01217 | 0.00446 | 0.22817 | 0.00623 | 0.00749 | 0.20380 | n.d. | n.d. | n.d. |
| 16329 |  | **M121** | AMS | **r** | 0.01549 | 0.00528 | 0.26819 | 0.00389 | 0.00499 | 0.12183 | n.d. | n.d. | n.d. |
| 16329 |  | **M124** | AMS | **r** | 0.01183 | 0.00488 | 0.26045 | 0.00350 | 0.01440 | 0.13432 | n.d. | n.d. | n.d. |
| 16329 |  | **M122** | AMS | **s** | 0.01557 | 0.00496 | 0.28124 | 0.00415 | 0.00295 | 0.09134 | n.d. | n.d. | n.d. |
| 16329 |  | **M123** | AMS | **s** | 0.01669 | 0.00233 | 0.33919 | 0.00241 | 0.00715 | 0.04739 | n.d. | n.d. | n.d. |
| 16406 | A | **M176** | AMS | **r** | 0.00471 | 0.01201 | 0.06006 | 0.01080 | 0.00100 | 0.43441 | n.d. | 0.00292 | 0.01821 |
| 16406 | B | **M173** | AMS | **r** | 0.00540 | 0.01229 | 0.22851 | 0.00634 | 0.00096 | 0.25698 | n.d. | 0.00372 | n.d. |
| 16406 | B | **M174** | AMS | **r** | 0.00404 | 0.01260 | 0.18217 | 0.00504 | 0.00179 | 0.34363 | n.d. | 0.00331 | n.d. |
| 16406 | A | **M177** | AMS | **s** | 0.00653 | 0.01101 | 0.27348 | 0.01916 | 0.00324 | 0.19105 | n.d. | 0.00395 | 0.02189 |
| 16607 | A | **M179** | SMS | **r** | 0.00760 | 0.00714 | 0.33079 | 0.00586 | 0.00351 | 0.16471 | n.d. | n.d. | n.d. |
| 16607 | A | **M181** | SMS | **r** | 0.00442 | 0.00009 | 0.39725 | 0.00237 | 0.00333 | 0.23231 | n.d. | n.d. | n.d. |
| 16607 | A | **M180** | SMS | **s** | 0.00831 | 0.00782 | 0.37967 | 0.00522 | 0.00167 | 0.09107 | n.d. | n.d. | n.d. |
| 16735 |  | **M108** | SMS | **r** | 0.00467 | 0.00159 | 0.08751 | 0.00199 | 0.01088 | 0.54186 | 0.00454 | n.d. | n.d. |
| 16735 |  | **M109** | SMS | **r** | 0.00343 | 0.00122 | 0.04622 | 0.00139 | 0.01188 | 0.60809 | 0.00606 | n.d. | n.d. |
| 16735 |  | **M110** | SMS | **r** | 0.01328 | 0.00382 | 0.27809 | 0.00290 | 0.00443 | 0.15191 | 0.00100 | n.d. | n.d. |
| 16735 |  | **M111** | SMS | **r** | 0.00587 | 0.00210 | 0.09414 | 0.00248 | 0.01022 | 0.48456 | 0.00469 | n.d. | n.d. |
| 16735 |  | **M112** | SMS | **s** | 0.01178 | 0.00546 | 0.22472 | 0.00434 | 0.00419 | 0.13719 | n.d. | n.d. | n.d. |
| 16753 |  | **M135** | SMS | **r** | 0.00334 | 0.00274 | 0.35394 | 0.00459 | 0.00314 | 0.20929 | n.d. | n.d. | n.d. |
| 16753 |  | **M136** | SMS | **r** | 0.00341 | 0.00323 | 0.40730 | 0.00463 | 0.00311 | 0.14022 | n.d. | n.d. | n.d. |
| 16753 |  | **M137** | SMS | **s** | 0.00290 | 0.00167 | 0.49044 | 0.00309 | 0.00404 | 0.07182 | n.d. | n.d. | n.d. |
| 17111 |  | **M283** | US | **r** | 0.00566 | 0.00452 | 0.41433 | 0.00503 | 0.00140 | 0.15528 | n.d. | n.d. | n.d. |
| 17111 |  | **M284** | US | **r** | 0.00312 | 0.00555 | 0.21009 | 0.00379 | 0.00228 | 0.43835 | n.d. | n.d. | n.d. |
| 17111 |  | **M285** | US | **s** | 0.00533 | 0.00510 | 0.48754 | 0.00315 | 0.00148 | 0.07089 | n.d. | n.d. | n.d. |
| 17251 |  | **M146** | US | **r** | 0.00499 | 0.00476 | 0.34576 | 0.00416 | 0.00586 | 0.22348 | n.d. | n.d. | n.d. |
| 17251 |  | **M147** | US | **s** | 0.00580 | 0.00099 | 0.46508 | 0.00408 | 0.00345 | 0.05957 | n.d. | n.d. | n.d. |
| 17279 |  | **M150** | US | **r** | 0.00627 | 0.00008 | 0.36987 | 0.00186 | 0.00337 | 0.15327 | n.d. | n.d. | n.d. |
| 17279 |  | **M152** | US | **s** | 0.00531 | 0.00157 | 0.50981 | 0.00458 | 0.00175 | 0.02050 | n.d. | n.d. | n.d. |
| 17316 |  | **M286** | US | **r** | 0.00275 | 0.00269 | 0.31869 | 0.00191 | 0.00142 | 0.35326 | n.d. | 0.00298 | n.d. |
| 17316 |  | **M287** | US | **s** | 0.00574 | 0.00661 | 0.53941 | 0.00281 | 0.00131 | 0.05459 | n.d. | 0.00482 | n.d. |
| 17318 |  | **M288** | US | **r** | 0.00290 | 0.00280 | 0.17769 | 0.00292 | 0.00085 | 0.50578 | n.d. | n.d. | n.d. |
| 17318 |  | **M289** | US | **s** | 0.00639 | 0.00602 | 0.40511 | 0.00628 | 0.00207 | 0.12189 | n.d. | n.d. | n.d. |
| 17375 | A | **M185** | US | **r** | 0.00211 | 0.00021 | 0.48274 | 0.00239 | 0.00789 | 0.14690 | n.d. | n.d. | n.d. |
| 17375 | A | **M186** | US | **r** | 0.00263 | 0.00124 | 0.42576 | 0.00298 | 0.00172 | 0.17270 | n.d. | n.d. | n.d. |
| 17375 | B | **M188** | US | **r** | 0.00142 | 0.00002 | 0.28553 | 0.00131 | 0.00072 | 0.41456 | n.d. | n.d. | n.d. |
| 17375 | B | **M189** | US | **r** | 0.00245 | 0.00033 | 0.42998 | 0.00140 | 0.00063 | 0.20994 | n.d. | n.d. | n.d. |
| 17375 | A | **M190** | US | **s** | 0.00313 | 0.00124 | 0.54562 | 0.00215 | 0.00176 | 0.03428 | n.d. | n.d. | n.d. |
| 17416 |  | **M294** | US | **r** | 0.00554 | 0.00116 | 0.45063 | 0.00260 | 0.00207 | 0.09138 | n.d. | n.d. | n.d. |
| 17416 |  | **M295** | US | **s** | 0.00523 | 0.00028 | 0.41963 | 0.00289 | 0.00191 | 0.06643 | n.d. | n.d. | n.d. |
| 17419 |  | **M292** | US | **r** | 0.00476 | 0.00158 | 0.39875 | 0.00379 | 0.00417 | 0.13693 | n.d. | n.d. | n.d. |
| 17419 |  | **M293** | US | **s** | 0.00558 | 0.00031 | 0.45210 | 0.00379 | 0.00767 | 0.07648 | n.d. | n.d. | n.d. |
| 17420 |  | **M290** | US | **r** | 0.00518 | 0.00140 | 0.39530 | 0.00350 | 0.00182 | 0.15180 | n.d. | n.d. | n.d. |
| 17420 |  | **M291** | US | **s** | 0.00433 | 0.00167 | 0.45588 | 0.00327 | 0.00298 | 0.04651 | n.d. | n.d. | n.d. |
| 17617 | B | **M192** | US | **r** | 0.00309 | 0.00418 | 0.29945 | 0.00219 | 0.00111 | 0.27638 | n.d. | n.d. | n.d. |
| 17617 | B | **M194** | US | **s** | 0.00433 | 0.00827 | 0.40065 | 0.00214 | 0.00163 | 0.10506 | n.d. | n.d. | n.d. |
| 17740 |  | **M296** | SG-I | **r** | 0.00234 | 0.00117 | 0.25074 | 0.00199 | 0.01032 | 0.38327 | n.d. | n.d. | n.d. |
| 17740 |  | **M297** | SG-I | **s** | 0.00579 | 0.00097 | 0.45574 | 0.00300 | 0.00613 | 0.05147 | n.d. | n.d. | n.d. |
| 17787 |  | **M301** | SG-I | **r** | 0.00238 | 0.00177 | 0.11497 | 0.00283 | 0.00430 | 0.58150 | n.d. | n.d. | n.d. |
| 17787 |  | **M302** | SG-I | **s** | 0.00702 | 0.00381 | 0.30345 | 0.00707 | 0.00452 | 0.13880 | n.d. | n.d. | n.d. |
| 17828 |  | **M281** | SG-I | **r** | 0.00531 | 0.00296 | 0.39011 | 0.00314 | 0.00708 | 0.11977 | n.d. | n.d. | n.d. |
| 17828 |  | **M282** | SG-I | **s** | 0.00902 | 0.00179 | 0.33732 | 0.00328 | 0.00202 | 0.05544 | n.d. | n.d. | n.d. |
| 17847 |  | **M278** | SG-I | **r** | 0.00833 | 0.01427 | 0.09569 | 0.00386 | 0.00128 | 0.37978 | n.d. | 0.00488 | 0.00228 |
| 17847 |  | **M279** | SG-I | **r** | 0.00426 | 0.00697 | 0.03565 | 0.00404 | 0.00254 | 0.62455 | n.d. | 0.00157 | 0.00137 |
| 17847 |  | **M280** | SG-I | **s** | 0.01419 | 0.01169 | 0.18411 | 0.00590 | 0.00194 | 0.12378 | n.d. | 0.00756 | 0.00803 |
| 17956 | A | **M305** | SG-I | **r** | 0.00254 | 0.00181 | 0.15821 | 0.00296 | 0.02176 | 0.41660 | n.d. | n.d. | n.d. |
| 17956 | B | **M307** | SG-I | **r** | 0.00120 | 0.00211 | 0.01210 | 0.00253 | 0.00530 | 0.77480 | n.d. | n.d. | n.d. |
| 17956 | A | **M308** | SG-I | **s** | 0.00569 | 0.00640 | 0.45411 | 0.00412 | 0.00529 | 0.10050 | n.d. | n.d. | n.d. |
| 17960 |  | **M303** | SG-I | **r** | 0.00535 | 0.01551 | 0.06536 | 0.01068 | 0.00581 | 0.47161 | n.d. | n.d. | 0.00341 |
| 17960 |  | **M304** | SG-I | **s** | 0.00672 | 0.01653 | 0.08062 | 0.01066 | 0.00326 | 0.43077 | n.d. | n.d. | 0.00345 |
| 18007 |  | **M274** | SG-I | **r** | 0.00358 | 0.00374 | 0.32774 | 0.00337 | 0.00318 | 0.30560 | n.d. | n.d. | n.d. |
| 18007 |  | **M275** | SG-I | **s** | 0.00536 | 0.00024 | 0.49482 | 0.00254 | 0.00186 | 0.03716 | n.d. | n.d. | n.d. |
| 18009 | A | **M271** | SG-I | **r** | 0.00418 | 0.00176 | 0.02068 | 0.00296 | 0.01008 | 0.60739 | n.d. | 0.00416 | n.d. |
| 18009 | A | **M273** | SG-I | **s** | 0.01314 | 0.00485 | 0.06134 | 0.00621 | 0.00711 | 0.16074 | n.d. | 0.00596 | n.d. |
| 18127 |  | **M262** | SG-II | **r** | 0.00336 | 0.00521 | 0.37178 | 0.00348 | 0.00850 | 0.19968 | n.d. | n.d. | n.d. |
| 18127 |  | **M263** | SG-II | **s** | 0.00489 | 0.00567 | 0.44736 | 0.00422 | 0.00423 | 0.08687 | n.d. | n.d. | n.d. |
| 18227 |  | **M260** | SG-II | **r** | 0.00217 | 0.00172 | 0.18895 | 0.00250 | 0.00687 | 0.51050 | n.d. | n.d. | n.d. |
| 18227 |  | **M261** | SG-II | **s** | 0.00530 | 0.00046 | 0.40681 | 0.00296 | 0.00586 | 0.07241 | n.d. | n.d. | n.d. |
| 18465 |  | **M170** | SG-III | **r** | 0.00417 | 0.00183 | 0.43149 | 0.00293 | 0.00196 | 0.18616 | n.d. | n.d. | n.d. |
| 18465 |  | **M171** | SG-III | **s** | 0.00549 | 0.00168 | 0.53840 | 0.00143 | 0.00183 | 0.02896 | n.d. | n.d. | n.d. |
| 18477 |  | **M166** | SG-III | **r** | 0.00435 | 0.00342 | 0.32200 | 0.00407 | 0.01872 | 0.27268 | n.d. | n.d. | n.d. |
| 18477 |  | **M167** | SG-III | **s** | 0.00357 | 0.00295 | 0.53427 | 0.00259 | 0.00298 | 0.05959 | n.d. | n.d. | n.d. |
| 18477 |  | **M168** | SG-III | **s** | 0.00522 | 0.00038 | 0.51066 | 0.00268 | 0.00350 | 0.03513 | n.d. | n.d. | n.d. |
| 18704 | A | **M085** | SG-III | **r** | 0.00653 | 0.00429 | 0.37097 | 0.00351 | 0.00690 | 0.08962 | n.d. | n.d. | n.d. |
| 18704 | A | **M086** | SG-III | **r** | 0.00496 | 0.00089 | 0.42884 | 0.00311 | n.d. | 0.04956 | n.d. | n.d. | n.d. |
| 18704 | A | **M087** | SG-III | **r** | 0.00499 | 0.00145 | 0.39697 | 0.00318 | n.d. | 0.04901 | n.d. | n.d. | n.d. |
| 18704 | A | **M088** | SG-III | **r** | 0.00566 | 0.00722 | 0.32757 | 0.00496 | 0.02764 | 0.12087 | n.d. | n.d. | n.d. |
| 18704 | A | **M090** | SG-III | **r** | 0.00540 | 0.00705 | 0.36352 | 0.00393 | 0.00969 | 0.10510 | n.d. | n.d. | n.d. |
| 18704 | A | **M089** | SG-III | **s** | 0.00480 | 0.00013 | 0.42734 | 0.00264 | n.d. | 0.02526 | n.d. | n.d. | n.d. |
| 18704 | A | **M091** | SG-III | **s** | 0.00555 | 0.00023 | 0.46021 | 0.00096 | n.d. | 0.02917 | n.d. | n.d. | n.d. |
| 18704 | A | **M092** | SG-III | **s** | 0.00591 | 0.00119 | 0.42103 | 0.00324 | n.d. | 0.05745 | n.d. | n.d. | n.d. |
| 18705 |  | **M093** | SG-III | **r** | 0.00419 | 0.00131 | 0.38270 | 0.00195 | 0.00429 | 0.11263 | n.d. | n.d. | n.d. |
| 18705 |  | **M094** | SG-III | **r** | 0.00536 | 0.00030 | 0.43652 | 0.00241 | 0.00174 | 0.06488 | n.d. | n.d. | n.d. |
| 18705 |  | **M095** | SG-III | **r** | 0.00460 | 0.00831 | 0.36898 | 0.00235 | 0.00843 | 0.12825 | n.d. | n.d. | n.d. |
| 18705 |  | **M096** | SG-III | **r** | 0.00426 | 0.00715 | 0.41089 | 0.00070 | 0.00245 | 0.05897 | n.d. | n.d. | n.d. |
| 18705 |  | **M097** | SG-III | **r** | 0.00624 | 0.00781 | 0.37185 | 0.00076 | 0.00069 | 0.07863 | n.d. | n.d. | n.d. |
| 18705 |  | **M098** | SG-III | **r** | 0.00700 | 0.00692 | 0.38253 | 0.00153 | 0.00124 | 0.06350 | n.d. | n.d. | n.d. |
| 18705 |  | **M099** | SG-III | **r** | 0.00969 | 0.00678 | 0.36411 | 0.00131 | 0.00578 | 0.08607 | n.d. | n.d. | n.d. |
| 18705 |  | **M102** | SG-III | **r** | 0.00756 | 0.00622 | 0.30550 | 0.00180 | 0.00198 | 0.14053 | n.d. | n.d. | n.d. |
| 18705 |  | **M103** | SG-III | **r** | 0.00896 | 0.00489 | 0.31213 | 0.00212 | 0.00204 | 0.13333 | n.d. | n.d. | n.d. |
| 18705 |  | **M104** | SG-III | **r** | 0.00751 | 0.00505 | 0.38391 | 0.00083 | 0.00051 | 0.07928 | n.d. | n.d. | n.d. |
| 18705 |  | **M105** | SG-III | **r** | 0.00793 | 0.00775 | 0.36455 | 0.00154 | 0.00180 | 0.08485 | n.d. | n.d. | n.d. |
| 18705 |  | **M100** | SG-III | **s** | 0.00983 | 0.00610 | 0.40948 | 0.00163 | 0.00333 | 0.02410 | n.d. | n.d. | n.d. |
| 18705 |  | **M101** | SG-III | **s** | 0.00891 | 0.00451 | 0.42070 | 0.00182 | 0.00229 | 0.02127 | n.d. | n.d. | n.d. |
| 18705 |  | **M106** | SG-III | **s** | 0.00876 | 0.00413 | 0.43910 | 0.00093 | 0.00080 | 0.01842 | n.d. | n.d. | n.d. |
| 18705 |  | **M107** | SG-III | **s** | 0.00536 | 0.00121 | 0.47931 | 0.00118 | 0.00163 | 0.03175 | n.d. | n.d. | n.d. |
| 18716 |  | **M257** | SG-III | **r** | 0.00304 | 0.00436 | 0.18368 | 0.00567 | 0.00264 | 0.49921 | n.d. | n.d. | n.d. |
| 18716 |  | **M258** | SG-III | **s** | 0.00744 | 0.00324 | 0.32791 | 0.00821 | 0.00362 | 0.12908 | n.d. | n.d. | n.d. |
| 18716 |  | **M259** | SG-III | **s** | 0.00752 | 0.00839 | 0.37566 | 0.00630 | 0.01073 | 0.13498 | n.d. | n.d. | n.d. |
| 18728 | A | **M253** | SG-III | **r** | 0.00461 | 0.00531 | 0.42881 | 0.00273 | 0.00261 | 0.11989 | n.d. | n.d. | n.d. |
| 18728 | A | **M254** | SG-III | **r** | 0.00519 | 0.00792 | 0.32927 | 0.00391 | 0.00818 | 0.24248 | n.d. | n.d. | n.d. |
| 18728 | A | **M255** | SG-III | **s** | 0.00524 | 0.00578 | 0.44514 | 0.00324 | 0.00368 | 0.08555 | n.d. | n.d. | n.d. |
| 18728 | A | **M256** | SG-III | **s** | 0.00339 | 0.00028 | 0.53057 | 0.00160 | 0.00133 | 0.04084 | n.d. | n.d. | n.d. |
| 18788 | A | **M199** | SG-III | **r** | 0.00416 | 0.00055 | 0.41070 | 0.00218 | 0.00889 | 0.19962 | n.d. | n.d. | n.d. |
| 18788 | A | **M200** | SG-III | **r** | 0.00482 | 0.00450 | 0.38936 | 0.00375 | 0.01780 | 0.14508 | n.d. | n.d. | n.d. |
| 18788 | A | **M201** | SG-III | **r** | 0.00491 | 0.00240 | 0.43034 | 0.00421 | 0.00318 | 0.12545 | n.d. | n.d. | n.d. |
| 18788 | B | **M203** | SG-III | **r** | 0.00640 | 0.00230 | 0.38551 | 0.00384 | 0.00223 | 0.12698 | n.d. | n.d. | n.d. |
| 18788 | A | **M202** | SG-III | **s** | 0.00656 | 0.00132 | 0.41463 | 0.00416 | n.d. | 0.05733 | n.d. | n.d. | n.d. |
| 18788 | B | **M204** | SG-III | **s** | 0.00420 | 0.00136 | 0.43994 | 0.00307 | 0.00303 | 0.05669 | n.d. | n.d. | n.d. |
| 18700 |  | **M264** | SG-II:SG-III | **r** | 0.00444 | 0.00390 | 0.32678 | 0.00603 | 0.01086 | 0.24593 | n.d. | n.d. | n.d. |
| 18700 |  | **M265** | SG-II:SG-III | **r** | 0.00358 | 0.00346 | 0.30826 | 0.00424 | 0.00910 | 0.30550 | n.d. | n.d. | n.d. |
| 18700 |  | **M266** | SG-II:SG-III | **s** | 0.00629 | 0.00538 | 0.38598 | 0.00753 | 0.00794 | 0.14201 | n.d. | n.d. | n.d. |
| 18702 |  | **M195** | SG-II:SG-III | **r** | 0.00287 | 0.00419 | 0.15986 | 0.00290 | 0.00552 | 0.52669 | n.d. | n.d. | n.d. |
| 18702 |  | **M196** | SG-II:SG-III | **r** | 0.00371 | 0.00371 | 0.21339 | 0.00319 | 0.00444 | 0.40771 | n.d. | n.d. | n.d. |
| 18702 |  | **M197** | SG-II:SG-III | **s** | 0.00691 | 0.00353 | 0.48136 | 0.00235 | 0.00321 | 0.05016 | n.d. | n.d. | n.d. |
| 18702 |  | **M198** | SG-II:SG-III | **s** | 0.00856 | 0.00400 | 0.41647 | 0.00388 | 0.00788 | 0.06221 | n.d. | n.d. | n.d. |
| 18879 |  | **M207** | AMa | **r** | 0.00122 | 0.00131 | 0.08850 | 0.00224 | 0.00077 | 0.67600 | n.d. | n.d. | n.d. |
| 18879 |  | **M208** | AMa | **s** | 0.00574 | 0.00428 | 0.47943 | 0.00266 | 0.00128 | 0.07244 | n.d. | n.d. | n.d. |
| 18880 |  | **M205** | AMa | **r** | 0.00347 | 0.00553 | 0.22518 | 0.00500 | 0.00213 | 0.38716 | n.d. | n.d. | n.d. |
| 18880 |  | **M206** | AMa | **s** | 0.00557 | 0.00581 | 0.41802 | 0.00540 | 0.00195 | 0.09320 | n.d. | n.d. | n.d. |
| 18885 | B | **M210** | AMa | **r** | 0.00362 | 0.00570 | 0.19806 | 0.00472 | 0.00142 | 0.41023 | n.d. | n.d. | n.d. |
| 18885 | B | **M212** | AMa | **s** | 0.00718 | 0.00600 | 0.38995 | 0.00617 | 0.00096 | 0.08158 | n.d. | n.d. | n.d. |
| 18935 | A | **M213** | AMa | **r** | 0.00354 | 0.00448 | 0.41025 | 0.00160 | 0.00173 | 0.21403 | n.d. | 0.02089 | n.d. |
| 18935 | A | **M214** | AMa | **r** | 0.00307 | 0.00255 | 0.43672 | 0.00137 | 0.00126 | 0.18822 | n.d. | 0.02387 | n.d. |
| 18935 | A | **M215** | AMa | **s** | 0.00473 | 0.00454 | 0.55390 | 0.00100 | 0.00127 | 0.03200 | n.d. | 0.02510 | n.d. |
| 18935 | A | **M216** | AMa | **s** | 0.00483 | 0.00628 | 0.51541 | 0.00191 | 0.00126 | 0.03672 | n.d. | 0.02566 | n.d. |
| 18938 | B | **M217** | AMa | **r** | 0.00342 | 0.00326 | 0.27819 | 0.00502 | 0.00836 | 0.32119 | n.d. | n.d. | n.d. |
| 18938 | B | **M218** | AMa | **s** | 0.00641 | 0.00596 | 0.41144 | 0.00610 | 0.00568 | 0.11624 | n.d. | n.d. | n.d. |
| 19336 |  | **M251** | AMb | **r** | 0.00528 | 0.00381 | 0.34001 | 0.00330 | 0.00546 | 0.18675 | n.d. | n.d. | n.d. |
| 19336 |  | **M252** | AMb | **s** | 0.00554 | 0.00583 | 0.43420 | 0.00483 | 0.00380 | 0.09442 | n.d. | n.d. | n.d. |
| 19433 |  | **M249** | AMb | **r** | 0.00405 | 0.00589 | 0.20951 | 0.00550 | 0.00278 | 0.39787 | n.d. | n.d. | n.d. |
| 19433 |  | **M250** | AMb | **s** | 0.00688 | 0.00196 | 0.45409 | 0.00438 | 0.00191 | 0.06621 | n.d. | n.d. | n.d. |
| 19650 | B | **M238** | AMb | **r** | 0.00402 | 0.00482 | 0.44641 | 0.00303 | 0.00172 | 0.14484 | n.d. | n.d. | n.d. |
| 19650 | B | **M239** | AMb | **s** | 0.00602 | 0.00658 | 0.45892 | 0.00423 | 0.00333 | 0.08036 | n.d. | n.d. | n.d. |
| 19679 | A | **M240** | AMb | **r** | 0.00415 | 0.00588 | 0.23318 | 0.00366 | 0.00034 | 0.38232 | n.d. | n.d. | n.d. |
| 19679 | A | **M241** | AMb | **s** | 0.00531 | 0.00109 | 0.48204 | 0.00216 | 0.00069 | 0.04386 | n.d. | n.d. | n.d. |
| 19683 | B | **M242** | AMb | **r** | 0.00377 | 0.00417 | 0.47727 | 0.00315 | 0.00147 | 0.10213 | n.d. | n.d. | n.d. |
| 19683 | B | **M243** | AMb | **s** | 0.00452 | 0.00221 | 0.48306 | 0.00153 | 0.00311 | 0.03582 | n.d. | n.d. | n.d. |
| 19857 | B | **M247** | UM | **r** | 0.00461 | 0.00605 | 0.28942 | 0.00556 | 0.00097 | 0.27035 | n.d. | n.d. | n.d. |
| 19857 | B | **M248** | UM | **s** | 0.00500 | 0.00415 | 0.43274 | 0.00436 | 0.00190 | 0.06602 | n.d. | n.d. | n.d. |
| 19864 |  | **M244** | UM | **r** | 0.00240 | 0.00706 | 0.05318 | 0.00460 | 0.00262 | 0.66745 | n.d. | 0.00361 | n.d. |
| 19864 |  | **M245** | UM | **r** | 0.00323 | 0.00772 | 0.09242 | 0.00614 | 0.00199 | 0.54909 | n.d. | 0.00351 | n.d. |
| 19864 |  | **M246** | UM | **s** | 0.00440 | 0.00142 | 0.47519 | 0.00426 | 0.00161 | 0.08576 | n.d. | 0.00581 | n.d. |
| 20003 |  | **M225** | UM | **r** | 0.00302 | 0.00130 | 0.30789 | 0.00160 | 0.00257 | 0.32679 | n.d. | n.d. | n.d. |
| 20003 |  | **M226** | UM | **r** | 0.00344 | 0.00234 | 0.17866 | 0.00359 | 0.00207 | 0.48580 | n.d. | n.d. | n.d. |
| 20003 |  | **M227** | UM | **s** | 0.00710 | 0.00754 | 0.33178 | 0.00761 | 0.00399 | 0.09344 | n.d. | n.d. | n.d. |
| 20004 | A | **M228** | UM | **r** | 0.00301 | 0.00185 | 0.18000 | 0.00351 | 0.02689 | 0.41911 | n.d. | n.d. | n.d. |
| 20004 | B | **M231** | UM | **r** | 0.00383 | 0.00001 | 0.32147 | 0.00175 | 0.00370 | 0.28771 | n.d. | n.d. | n.d. |
| 20004 | A | **M229** | UM | **s** | 0.00609 | 0.00147 | 0.39339 | 0.00399 | 0.00381 | 0.07465 | n.d. | n.d. | n.d. |
| 20004 | B | **M232** | UM | **s** | 0.00558 | 0.00008 | 0.47294 | 0.00179 | 0.00767 | 0.05026 | n.d. | n.d. | n.d. |
| 20004 | B | **M233** | UM | **s** | 0.00739 | 0.00234 | 0.39933 | 0.00341 | 0.00845 | 0.08156 | n.d. | n.d. | n.d. |
| 20045 |  | **M222** | UM | **r** | 0.00706 | 0.00082 | 0.43026 | 0.00372 | 0.00267 | 0.09983 | n.d. | n.d. | n.d. |
| 20045 |  | **M223** | UM | **r** | 0.00618 | 0.00224 | 0.40911 | 0.00474 | 0.00264 | 0.11228 | n.d. | n.d. | n.d. |
| 20045 |  | **M224** | UM | **s** | 0.00707 | 0.00145 | 0.45708 | 0.00373 | 0.00354 | 0.06016 | n.d. | n.d. | n.d. |
| 20345 | A | **M157** | UM | **r** | 0.00844 | 0.00182 | 0.46460 | 0.00261 | 0.00246 | 0.06164 | n.d. | n.d. | n.d. |
| 20345 | A | **M158** | UM | **r** | 0.00799 | 0.00349 | 0.43870 | 0.00382 | 0.00380 | 0.08414 | n.d. | n.d. | n.d. |
| 20345 | B | **M161** | UM | **r** | 0.00658 | 0.00290 | 0.42729 | 0.00377 | 0.00240 | 0.07618 | n.d. | n.d. | n.d. |
| 20345 | A | **M160** | UM | **s** | 0.00765 | 0.00017 | 0.50343 | 0.00228 | 0.00131 | 0.02950 | n.d. | n.d. | n.d. |
| 20345 | B | **M163** | UM | **s** | 0.00662 | 0.00263 | 0.45333 | 0.00316 | 0.00243 | 0.06139 | n.d. | n.d. | n.d. |

**LS: Lower Solutrean (21000-20500 BP). AMS and SMS: Ancient and Recent Middle Solutrean (20500-20000 BP). US: Upper Solutrean (2000-19500 BP). SG-I, SGII and SGIII: Solutreo-Gravettian I, II and III (19500-17000 BP). AM: Ancient Magdalenian (17000-145000 BP). UM: Midlde and Upper Magdalenian (14.500-12.000). Chronologies are only indicatives.**
