# Supplementary material for: A Unique Collection of Palaeolithic Painted Portable Art: Characterization of Red and Yellow Pigments from the Parpalló Cave (Spain)
Source: PLoS One. 2016 Oct 12;11(10):e0163565. doi: 10.1371/journal.pone.0163565 (PMC5061316; doi:10.1371/journal.pone.0163565)
Supplement: S4 Table — (n.d. = non detected). (DOCX) [file pone.0163565.s004.docx]

S4_Supporting_Information_Table. Normalized net areas of the elements detected by EDXRF in yellow (y) motifs and rock supports (s) from the Parpalló plaquettes. (n.d.= non detected).

| Plaquette # | Face | Code | Period | Color | **Si** | **K** | **Ca** | **Ti** | **Mn** | **Fe** | **Pb** | **Sr** | **Zr** |
| --- | --- | --- | --- | --- | --- | --- | --- | --- | --- | --- | --- | --- | --- |
| 16406 | A | **M175** | AMS | **y** | 0.00457 | 0.01284 | 0.05255 | 0.00929 | 0.00051 | 0.24871 | n.d. | 0.00160 | 0.01021 |
| 16406 |  | **M177** | AMS | **s** | 0.00625 | 0.01187 | 0.23566 | 0.01633 | 0.00130 | 0.10730 | n.d. | 0.00200 | 0.01153 |
| 16607 | A | **M178** | SMS | **y** | 0.00434 | 0.00206 | 0.22601 | 0.00282 | 0.00094 | 0.18623 | n.d. | n.d. | n.d. |
| 16607 | B | **M182** | SMS | **y** | 0.00370 | 0.00130 | 0.29524 | 0.00206 | 0.00378 | 0.16317 | n.d. | n.d. | n.d. |
| 16607 | B | **M183** | SMS | **y** | 0.00305 | 0.00101 | 0.29402 | 0.00207 | 0.00239 | 0.18062 | n.d. | n.d. | n.d. |
| 16607 | A | **M180** | SMS | **s** | 0.00820 | 0.00170 | 0.33361 | 0.00432 | 0.00083 | 0.05218 | n.d. | n.d. | n.d. |
| 16607 | B | **M184** | SMS | **s** | 0.00420 | 0.00127 | 0.39657 | 0.00338 | 0.00334 | 0.05520 | n.d. | n.d. | n.d. |
| 17375 | A | **M187** | US | **y** | 0.00132 | 0.00321 | 0.17027 | 0.00230 | 0.00030 | 0.30037 | n.d. | n.d. | n.d. |
| 17375 |  | **M190** | US | **s** | 0.00305 | 0.00510 | 0.47545 | 0.00188 | 0.00094 | 0.02022 | n.d. | n.d. | n.d. |
| 17617 | B | **M191** | US | **y** | 0.00153 | 0.00451 | 0.14577 | 0.00173 | 0.00117 | 0.28651 | n.d. | n.d. | n.d. |
| 17617 |  | **M193** | US | **y** | 0.00272 | 0.00475 | 0.34532 | 0.00180 | 0.00071 | 0.08066 | n.d. | n.d. | n.d. |
| 17617 | B | **M194** | US | **s** | 0.00420 | 0.00982 | 0.34746 | 0.00198 | 0.00103 | 0.06006 | n.d. | n.d. | n.d. |
| 17742 | A | **M298** | SG-I | **y** | 0.00455 | 0.00651 | 0.31522 | 0.00339 | 0.00387 | 0.10519 | n.d. | n.d. | n.d. |
| 17742 | A | **M299** | SG-I | **y** | 0.00394 | 0.00572 | 0.25992 | 0.00371 | 0.00479 | 0.15906 | n.d. | n.d. | n.d. |
| 17742 | A | **M300** | SG-I | **s** | 0.00527 | 0.00660 | 0.36819 | 0.00424 | 0.00275 | 0.05061 | n.d. | n.d. | n.d. |
| 17956 | A | **M306** | SG-I | **y** | 0.00372 | 0.00505 | 0.23642 | 0.00276 | 0.00471 | 0.15720 | n.d. | n.d. | n.d. |
| 17956 | B | **M308** | SG-I | **s** | 0.00525 | 0.00803 | 0.39043 | 0.00353 | 0.00289 | 0.05765 | n.d. | n.d. | n.d. |
| 18005 |  | **M276** | SG-I | **y** | 0.00290 | 0.00617 | 0.18974 | 0.00262 | 0.00118 | 0.19905 | n.d. | n.d. | n.d. |
| 18005 |  | **M277** | SG-I | **s** | 0.00176 | 0.00537 | 0.39906 | 0.00072 | 0.00073 | 0.01287 | n.d. | n.d. | n.d. |
| 18009 | B | **M272** | SG-I | **y** | 0.00927 | 0.00462 | 0.01026 | 0.00473 | 0.00145 | 0.26833 | n.d. | 0.00339 | n.d. |
| 18009 |  | **M273** | SG-I | **s** | 0.01263 | 0.00859 | 0.05372 | 0.00533 | 0.00387 | 0.09153 | n.d. | 0.00331 | n.d. |
| 18037 |  | **M269** | SG-II | **y** | 0.00274 | 0.00237 | 0.18696 | 0.00166 | 0.00055 | 0.24853 | n.d. | n.d. | n.d. |
| 18037 |  | **M270** | SG-II | **s** | 0.00464 | 0.00216 | 0.40378 | 0.00084 | 0.00098 | 0.00981 | n.d. | n.d. | n.d. |
| 18206 |  | **M267** | SG-II | **y** | 0.00261 | 0.00214 | 0.33943 | 0.00335 | 0.00280 | 0.13515 | n.d. | n.d. | n.d. |
| 18206 |  | **M268** | SG-II | **s** | 0.00526 | 0.00162 | 0.45224 | 0.00211 | 0.00158 | 0.02825 | n.d. | n.d. | n.d. |
| 18465 |  | **M169** | SG-III | **y** | 0.00271 | 0.00112 | 0.07715 | 0.00278 | 0.00401 | 0.37033 | n.d. | n.d. | n.d. |
| 18465 |  | **M172** | SG-III | **y** | 0.00501 | 0.00179 | 0.43441 | 0.00236 | 0.00107 | 0.05545 | n.d. | n.d. | n.d. |
| 18465 |  | **M171** | SG-III | **s** | 0.00532 | 0.00157 | 0.46572 | 0.00123 | 0.00100 | 0.01660 | n.d. | n.d. | n.d. |
| 18477 |  | **M164** | SG-III | **y** | 0.00192 | 0.00457 | 0.09981 | 0.00222 | 0.00154 | 0.35407 | n.d. | n.d. | n.d. |
| 18477 |  | **M165** | SG-III | **y** | 0.00359 | 0.00135 | 0.27862 | 0.00318 | 0.00330 | 0.17036 | n.d. | n.d. | n.d. |
| 18477 | B | **M167** | SG-III | **s** | 0.00351 | 0.00294 | 0.46538 | 0.00224 | 0.00161 | 0.03398 | n.d. | n.d. | n.d. |
| 18477 |  | **M168** | SG-III | **s** | 0.00505 | 0.00162 | 0.44480 | 0.00234 | 0.00184 | 0.02106 | n.d. | n.d. | n.d. |
| 18885 | A | **M209** | AM | **y** | 0.00404 | 0.00141 | 0.19571 | 0.00458 | 0.00171 | 0.22364 | n.d. | n.d. | n.d. |
| 18885 | B | **M211** | AM | **y** | 0.00393 | 0.00186 | 0.15860 | 0.00556 | 0.00054 | 0.24288 | n.d. | n.d. | n.d. |
| 18885 | B | **M212** | AM | **s** | 0.00692 | 0.00831 | 0.33528 | 0.00528 | 0.00043 | 0.04646 | n.d. | n.d. | n.d. |
| 18885 | B | **M212** | AM | **s** | 0.00692 | 0.00831 | 0.33528 | 0.00528 | 0.00043 | 0.04646 | n.d. | n.d. | n.d. |
| 18929 |  | **M219** | AM | **y** | 0.00344 | 0.00151 | 0.16261 | 0.00330 | 0.00079 | 0.26166 | 0.00229 | n.d. | n.d. |
| 18929 |  | **M221** | AM | **y** | 0.00168 | 0.00124 | 0.07665 | 0.00274 | 0.00130 | 0.36009 | 0.00126 | n.d. | n.d. |
| 18929 |  | **M220** | AM | **s** | 0.00904 | 0.00297 | 0.31513 | 0.00318 | 0.00065 | 0.04763 | n.d. | n.d. | n.d. |

**LS: Lower Solutrean (21000-20500 BP). AMS and SMS: Ancient and Recent Middle Solutrean (20500-20000 BP). US: Upper Solutrean (2000-19500 BP). SG-I, SGII and SGIII: Solutreo-Gravettian I, II and III (19500-17000 BP). AM: Ancient Magdalenian (17000-145000 BP). UM: Midlde and Upper Magdalenian (14.500-12.000). Chronologies are only indicatives.**
